# An integrated reference atlas of human skeletal muscle

**DOI:** 10.1016/j.ebiom.2026.106465

**Published:** 2026-08-31

**Authors:** Christopher Nelke, Franziska Babilon, Christina B. Schroeter, Anne-Katrin Güttsches, Paula Quint, Karsten Krause, Gerd Meyer zu Hörste, Benedikt Schoser, Jörg H.W. Distler, Sven G. Meuth, Felix Kleefeld, Tobias Ruck

**Affiliations:** a Department of Neurology, Ruhr University Bochum, BG University Hospital Bergmannsheil, Bochum, Germany; b BG University Hospital Bergmannsheil, Heimer Institute for Muscle Research, Bochum, Germany; c Department of Neurology, Medical Faculty, Heinrich Heine University Duesseldorf; Duesseldorf, Germany; d Department of Neurology, University Hospital Münster, Münster, Germany; e Friedrich Baur Institute at the Department of Neurology, LMU University Hospital, LMU Munich, Munich, Germany; f Department for Rheumatology, University Hospital Düsseldorf, Medical Faculty of Heinrich Heine University, Duesseldorf, Germany; g Hiller Research Center, University Hospital Düsseldorf, Medical Faculty of Heinrich Heine University, Duesseldorf, Germany

**Keywords:** Muscle, Atlas, Transcriptomics, Reference, Single cell, Single nucleus

## Abstract

**Background:**

Single-cell and single-nucleus RNA sequencing have transformed our understanding of human skeletal muscle biology, yet reproducibility and cross-study comparison remain limited by the lack of a unified reference framework and consistent cell-type annotation.

**Methods:**

We systematically searched for scRNA-seq and snRNA-seq datasets from adult human skeletal muscle. Seven eligible studies were retrieved and harmonised. We benchmarked multiple integration strategies to construct a joint reference atlas and derived modality-aware marker panels. Selected findings were validated by immunofluorescence in muscle biopsies.

**Findings:**

We generated a harmonised atlas comprising 122,000 cells and 630,000 nuclei from 88 healthy individuals and resolved 17 major skeletal muscle cell populations, spanning mononuclear compartments and multinucleated myofibers. Cross-modality analysis identified tissue- and modality-aware marker panels and nominated both established and previously unrecognised markers. *NOVA1* emerged as a selective marker of fibro-adipogenic progenitors and was validated at the transcript and protein levels. Focusing on myonuclei, pseudotime modelling reconstructed differentiation trajectories from quiescent muscle stem cells to mature type I and type II myofibers and revealed lineage-specific programs, including transient activation of protocadherin-γ genes during type I myofiber differentiation. We further provide an interactive web application for marker-based cell-type prediction using the reference atlas.

**Interpretation:**

This integrated reference atlas and accompanying annotation tool establish a standardised framework for human muscle transcriptomics, promoting consistent cell-type assignment and providing a baseline for future studies of muscle development, ageing, and disease.

**Funding:**

Else Kröner-Fresenius-Stiftung and the German Research Foundation.

**Research in context**

**Evidence before this study**

We searched PubMed and Gene Expression Omnibus (GEO) from database inception to April 2026 to identify studies generating single-cell RNA sequencing (scRNA-seq) or single-nucleus RNA sequencing (snRNA-seq) data from adult human skeletal muscle tissue. Searches were not restricted by language. We used two query strings combining terms for single-cell or single-nucleus RNA-sequencing with “skeletal muscle”, “human”, and modality, and additionally screened reference lists and repository cross-links for eligible datasets. We included studies profiling healthy adult donors with publicly available raw or processed data, available as peer-reviewed publications or preprints, and excluded bulk RNA-seq, non-human tissue, and studies without accessible data. Across 485 records, seven studies met eligibility criteria. Collectively, existing muscle transcriptomic atlases provided important insights into muscle-resident cell populations and (in more recent work) myofiber nuclei and ageing-associated changes, but they remained fragmented by modality (cells versus nuclei), sample processing and platform differences, and non-unified annotation strategies, limiting direct cross-study comparability and reproducible cell-type assignment. Because the available evidence consisted of descriptive resource datasets rather than interventional effect estimates, meta-analysis was not applicable; instead, the main quality concerns were sampling heterogeneity and batch effects that complicate integration and consistent annotation.

**Added value of this study**

This study creates a harmonised, integrated reference atlas of human skeletal muscle by synthesising both scRNA-seq and snRNA-seq datasets into a single framework and scaling beyond any individual study, integrating nearly 122,000 cells and 630,000 nuclei from 88 donors. By jointly embedding across studies and modalities and systematically comparing integration strategies, we provide a standardised cell-type map. We further deliver modality-aware, muscle-specific marker panels derived from unbiased differential expression, enabling more reliable annotation across platforms. To make the resource broadly useable, we provide an interactive online annotation tool that assigns cell identity from user-defined marker lists and benchmark its performance against general-purpose automated annotation methods that lack skeletal muscle–specific references. Finally, we leverage the atlas to build a harmonised view of myogenic differentiation and to nominate and validate previously underappreciated lineage markers and differentiation programs, illustrating how large-scale integration can reveal biology that is difficult to detect in single datasets.

**Implications of all the available evidence**

Taken together with prior muscle transcriptomic studies, our findings support the need for a shared reference framework that allows investigators to place new datasets into a common taxonomy, report cell-type composition consistently, and compare cellular programs across studies, platforms, and cohorts. This atlas provides a practical foundation to accelerate discovery by enabling systematic mapping of disease and ageing datasets onto a healthy reference, strengthening cross-study reproducibility.

## Introduction

Single-cell and single-nucleus RNA sequencing (scRNA-seq and snRNA-seq) have advanced our understanding of skeletal muscle biology, providing unprecedented resolution of the cellular heterogeneity and the signalling networks within muscle tissue.^1^, ^2^, ^3^, ^4^, ^5^, ^6^ However, interpreting and reproducing findings from these high-dimensional datasets remains challenging due to the absence of a unified cell-type taxonomy and standardised reference framework.

In other organ systems, large-scale cell atlases have emerged as transformative resources for addressing these challenges. Initiatives such as the Human Cell Atlas and Tabula Sapiens have integrated millions of cells across diverse tissues, enabling cross-study harmonisation, unbiased marker discovery, and systematic mapping of cellular states and trajectories.^7^, ^8^, ^9^, ^10^ These atlases provide standardised cell-type definitions, facilitate direct comparison between studies, and serve as foundational scaffolds for interpreting new datasets within a common biological context.

Comparable large-scale efforts are now emerging in muscle research, exemplified by two recent landmark studies that generated high-resolution transcriptomic atlases of ageing human muscle.^1^^,^^2^ Both studies identified age-related changes in architectural and signalling networks, including pro-inflammatory and pro-fibrotic interaction circuits that likely contribute to frailty and functional decline.^1^^,^^2^ However, variation in annotation strategies and inconsistent reporting of cell-type compositions across these datasets illustrate the ongoing challenge posed by the lack of a harmonised reference framework in skeletal muscle research.

The next logical step for the field is to establish a consensus atlas that unites existing data and resolves annotation discrepancies. To address this need, we integrated 122,000 cells and 630,000 nuclei from publicly available datasets to generate a comprehensive atlas of human skeletal muscle. This resource provides a standardised reference framework for cell-type annotation, an online annotation tool enabling cell identity assignment from user-defined marker lists, and a detailed characterisation of the myogenic lineage architecture. Together, these deliverables are intended to harmonise muscle transcriptomic research, improve reproducibility across studies, and accelerate the discovery of new biological insights.

## Methods

### Ethics

The study was conducted in accordance with the Declaration of Helsinki. All patient samples included are covered by their respective Ethics committee approval. Patient sample analysis for this study was approved by the local Ethics committee of the Heinrich Heine University Düsseldorf, Germany (2016-053-f-S). This specifically applies for the data derived from Nelke et al., 2023 and the analysis of muscle specimen for this study using immunofluorescence.^4^ Informed consent was required and obtained from all patients involved. All other data are covered by the original Ethics committee approval of the respective study as detailed below:

Rubenstein et al., 2020: All procedures associated with the research were approved by the Institutional Review Board at Ball State University (IRB protocol 123578).

Micheli et al., 2020: All procedures were approved by the Institutional Review Board at Weill Cornell Medical College (WCMC IRB Protocol # 1510016712).

Osolkov et al., 2022: The study was approved by the local Ethics committee, Lund University (Dnr 2015/593).

Perez et al., 2022: All methods and procedures in this study were approved by the Hamilton Integrated Research Ethics Board (HIREB 2018-4656-GRA).

Kedlian et al., 2024: Ethical approval was granted by the Research Ethics Committee (REC) East of England–Cambridge South (REC ref. 15/EE/0152).

Lai et al., 2024: Ethical approval was granted for the European cohort by the Research Ethics Committee of Hospital Arnau de Vilanova (CEIm 28/2019), and for the Asian cohort by the Institutional Ethics Committee of the First Affiliated Hospital/School of Clinical Medicine of Guangdong Pharmaceutical University, Guangzhou (China) (2020-ICE-90).

The study was conducted from November 2024 to April 2026. Participants who provided muscle biopsies for immunofluorescence validation were recruited in December of 2025.

### Literature search and study eligibility

We conducted a systematic search of the literature and public repositories to identify studies that generated single-cell or single-nucleus RNA-sequencing data from adult human skeletal muscle. Searches were performed in PubMed and Gene Expression Omnibus (GEO) in April 2026 using the following search string: ((“single-cell RNA sequencing”[MeSH] OR “scRNA-seq” OR “single cell RNA-seq” OR “single-nucleus RNA sequencing” OR “snRNA-seq” OR “single nucleus RNA-seq”) AND (“skeletal muscle”[MeSH] OR “human skeletal muscle” OR “skeletal muscle tissue”) AND “humans”[MeSH] AND (“adult” OR “mature” OR “biopsy” OR “tissue”)). Studies were eligible for inclusion if they analysed adult human skeletal muscle tissue from healthy donors, generated scRNA-seq or snRNA-seq data, provided publicly available raw or processed data, and were reported in a peer-reviewed publication or deposited as a preprint. We excluded studies that were based exclusively on bulk RNA-seq, used in vitro-differentiated cells or animal tissue, did not include eligible adult human skeletal muscle samples, or lacked publicly available raw or processed data.

From an initial yield of 485 records, duplicates were removed and titles and abstracts screened independently by two reviewers (CN and FB), yielding 21 full-text articles for eligibility assessment. We extracted metadata from each included study, including donor demographics (age, sex) and experimental method (scRNA-seq or snRNA-seq, sequencing platform). Following a full review, 7 studies met all inclusion criteria and were selected for downstream integration and analysis. Metadata for all individual samples included in this study are provided in the Supplementary Table S1. We did not stratify by sex for this study. The reported sex from the original publication was used.

### Preprocessing and quality control of data

Fastq files were processed to gene expression matrices using CellRanger (v9.0) or CeleScope (v2.7). Briefly, fastq files were demultiplexed using cell barcodes and UMIs. Adaptor sequences and poly-A tails were trimmed and aligned to the GRCh38 version of the human genome with Ensembl version 92 gene annotations (STAR v2.7.2a). For all datasets, we applied the same quality control metrices. We excluded cells with a unique feature count below 200 or above 5000, or with a mitochondrial ratio exceeding 10% (Supplementary Fig. S1A and B). A combined expression matrix was constructed from all sequenced experiments for downstream analysis.

### Clustering, differential expression and enrichment analysis

All data were converted to an R-compatible format and processed using the Seurat pipeline (version 5.2.1).^11^ Nuclei and cells were filtered based on the quality metrics above. Data was normalised by natural-log transformation, and highly variable genes were calculated using the FindVariableFeatures() function with “vst” mode and the top 2000 features selected. The data were scaled, and principal component analysis (PCA) was performed for dimensionality reduction. To account for batch effects and integrate multiple datasets, we compared a total of three different algorithms: Harmony,^12^ Canonical correlation analysis (CCA)^11^ and Fast integration using reciprocal PCA (RPCA)^11^ were applied in the Seurat environment. For Harmony we applied a theta of 2.5. For RPCA, we compared different integration strengths with k.anchor set to 5, 10, or 20. The first 30 PCA dimensions were used for integration. We also tested single-cell variational inference (scVI) integration using the reticulate interface to Python.^13^ Given that raw data were unavailable for some datasets, the scVI integration yielded spurious results, and we did not use it further. Integrations were compared based on biological information and known marker genes by two investigators (CN and FB). Clustering was conducted using the Seurat pipeline with a resolution set to 0.8, and clusters were visualised using Uniform Manifold Approximation and Projection (UMAP). Cell types were annotated based on canonical marker genes. After manual annotation, we assessed the contribution of scRNA-seq and snRNA-seq to each annotated cell type. For cell types represented by both modalities in the integrated space, differential expression analysis was performed separately within each modality, using the shared cell-type labels.

To identify marker genes, we performed marker detection separately for single-cell and single-nucleus data. Within each modality, we applied Seurat’s FindMarkers function in a one-versus-rest design for each annotated cell type using the Wilcoxon rank-sum test. Only positive markers were considered, with a minimum expression fraction of 20% and a minimum average log2 fold-change of 0.25. For each marker, we calculated the difference in detection frequency between the target cell type and all other cells and the detection ratio. Genes were ranked using a specificity-aware marker score defined as: avg_log2FC × (pct.1 − pct.2) × log2((pct.1 + 0.05)/(pct.2 + 0.05)) × sqrt(pct.1). This ranking prioritises genes with high fold-change, broad detection within the target population, and low detection in the background population. For each cell type and modality, the top three markers were retained after filtering. The final marker-gene list was generated as the ordered union of the selected single-cell and single-nucleus markers, with duplicate genes retained only once.

### Regression analysis

We treated *NOVA1* expression as a continuous variable and cells were ordered and partitioned into 50 bins. For each gene, we computed the mean log-expression within each *NOVA1* bin. Gene inclusion required detection in ≥0.5% of FAPs and non-zero variance across bins.

Per-gene association with *NOVA1* was quantified by Pearson correlation between the gene’s bin-mean profile and the *NOVA1* bin means. Test statistics were derived as $t=r\;\sqrt{\left(\frac{B\;-\;2}{1\;-\;r^{2}}\right)}$ with df=B−2, and Benjamini–Hochberg FDR was used to control for multiple testing. Genes were ranked by the signed *t* statistic from the bin-mean regression. Pre-ranked enrichment was performed with fgseaMultilevel on the GO Biological Process database.

### Pseudotime inference and trajectory analysis

MF nuclei were extracted from the snRNA-seq dataset for trajectory analysis. Pseudotime trajectories were computed using the Palantir algorithm,^14^ which models cell differentiation and assigns pseudotime to individual nuclei based on diffusion maps. The log-normalised gene expression matrix was used as input, and pseudotime values were extracted for downstream comparative analysis. To quantitatively compare gene expression dynamics along pseudotime trajectories between type I and II MF, we applied the Genes2Genes framework.^15^ For each MF type, AnnData objects containing log-normalised gene expression and inferred pseudotime were generated. Pseudotime values were normalised to the 0; 1 range. The optimal number of discrete pseudotime bins was estimated using the optbinning package, and all subsequent trajectory alignments were performed using *n* = 14 bins.

Gene-level trajectory alignment was conducted with G2G’s RefQueryAligner, using highly variable genes as the input gene set. Dynamic programming alignment was used to match gene expression trajectories across pseudotime between both MF types, and alignment statistics, including optimal alignment cost and similarity percentage, were computed for each gene. Genes were ranked according to alignment similarity. Gene clusters were calculated using the ClusterUtils function after calculation of the optimal distance matrix (Levenshtein distance).

All code for the trajectory analysis was implemented in Python (genes2genes v0.2.0).

### Validation with immunofluorescence

Fresh-frozen biopsies were cryosectioned at 8 μm. Biopsies were analysed from patients with inclusion body myositis (IBM) and healthy controls (HC). IBM patients were required to meet the 2024 European Neuromuscular Centre (ENMC) diagnostic criteria.^16^ HC were required to have no abnormalities on the histopathological examination, as previously described.^4^^,^^17^ Sections were fixed in 4% paraformaldehyde (ThermoFisher, Catalogue number A11313.0E) for 10 min, rinsed twice in PBS (ThermoFisher, Catalogue number 10010023), washed 5 min in PBS, permeabilized in PBS with Triton X-100 (ThermoFisher, Catalogue number A16046.AE) for 5 min, rinsed twice in PBS, then washed 5 min in PBS with Tween-20 (ThermoFisher, Catalogue number J61544.K3). After two PBS rinses, nonspecific binding was blocked with 5% normal goat serum (NGS, ThermoFisher, Catalogue number 31872) in PBS for 30 min. Primary antibodies were applied overnight at 4 °C in blocking buffer: rabbit anti-NOVA1 (1:500, Invitrogen, #PA5-95571, RRID: AB_2807373), rabbit anti-DCN (1:200, ThermoFisher, #PA5-95830, RRID: AB_2807632) and mouse anti-PDGFRA (1:200, abcam, #ab96569, RRID: AB_10687154). The next day, sections were rinsed twice in PBS and washed 5 min in PBS, followed by incubation with species-appropriate secondary antibodies for 1.5 h at RT. These were Alpaca Anti-Mouse (Jackson ImmunoResearch, RRID AB_3095461) and Alpaca Anti-Rabbit (Jackson ImmunoResearch, RRID AB_2922867). Nuclei were counterstained with DAPI (ThermoFisher, Catalogue number D1306) for 5 min, sections were rinsed (2 × PBS, then 5 min PBS) and mounted in 70% glycerol in PBS. Images were acquired on an LSM 980 with Airyscan 2 confocal microscope (Zeiss). Acquisition settings were kept constant within each experiment. The quantification was performed in randomly distributed 10 high-power fields (HPF, based on the microscope used and the respective oculars ≙ 0.16 mm^2^) as previously described.^4^^,^^17^ The mean was calculated for each sample. Antibodies were validated as follows: Anti-DCN, anti-PDGFRA and anti-NOVA1 were validated by the absence of a specific staining in control sections (mouse spleen and mouse liver). The species specificity of the anti-mouse and anti-rabbit secondary antibodies was validated by omitting the corresponding species-specific primary antibody; no detectable signal was observed under these conditions.

### Cross-validation of protocadherin expression and protein levels

To cross-validate protocadherin (PCDH) gene expression, we re-analysed the RNA-seq dataset originally published by Moreno-Justicia et al.^18^ Raw sequencing data were obtained from the European Genome-phenome Archive (EGA) at EGAS50000000418 and processed following the authors’ pipeline. Briefly, reads were aligned to the human reference genome (GRCh38) using STAR (v2.6.0c), and resulting BAM files were indexed with SAMtools (v1.11). PCR duplicates were removed using umi_tools (v1.0.1). Gene-level quantification was performed with featureCounts from the Subread package (v2.0.3). Quality control was conducted using FastQC (v0.11.9). For downstream analysis, we generated pseudobulk expression profiles for each MF subtype within the 1000-fibre transcriptomics dataset. We specifically queried the dataset for all annotated *PCDH* genes and calculated their normalised expression levels comparing type I and type II MF, as described by the authors. Furthermore, we reanalysed the available proteomics data to compare type I and type II MF. For this purpose, we queried the Supplementary Dataset 10 of the original study, providing Pseudobulked protein abundance for both MF subtypes.

### Development of an online tool

For each cell type, marker genes were ranked by their average fold change. The top 500 genes per cell type were selected. This cutoff was chosen as a pragmatic balance between specificity and robustness. A web-based, interactive cell type prediction application was implemented using the R Shiny framework (shiny v1.8.1). To prioritise gene ranking in the prediction process, the input gene list is weighted such that the top gene receives the maximum score, with subsequent genes receiving incrementally lower weights. To account for the rank order of the submitted gene list, genes are assigned positional weights. For an input list containing N genes, the gene at rank i is assigned the weight wi = N − i + 1. Thus, the first gene receives the highest weight, and subsequent genes receive incrementally lower weights. For each reference cell type, the algorithm computes the number of user-supplied genes that overlap with the cell type’s top 500 reference markers and a weighted score, which is the sum of positional weights for overlapping genes, with higher weights assigned to matches found higher in the input list. Predicted cell types are ranked by weighted score (primary) and match count (secondary). The complete app and the marker gene reference file were deployed to shinyapps.io using the rsconnect package (v1.1.0) and standard cloud deployment workflows.

For external validation, the ranked marker gene lists provided from an independent snRNA-seq study^3^ were retrieved and used as query inputs. Each external marker list was scored against all atlas-derived reference cell types using the same weighting and ranking procedure implemented in the web application.

### Automated cell-type annotation and benchmarking

We benchmarked automated annotation tools—CHETAH,^19^ SingleR,^20^ and Azimuth pipelines^21^—against our manual label annotation. The full dataset was downsampled to 50,000 cells/nuclei and stratified by cell types. Analyses were performed on log-normalised counts, exported as a sparse SingleCellExperiment. For reference-based annotation, we used the Human Primary Cell Atlas (HPCA; celldex::HumanPrimaryCellAtlasData). Query and reference were gene-matched by intersecting symbols and re-ordering rows to identical gene order. SingleR was run in serial mode with de.method = “wilcox” and pruning enabled (prune = TRUE), assigning low-confidence assignments to *Unassigned*. CHETAH was run with the default settings. Within each manual class and method, rare predicted labels were collapsed into “Other”.

### Statistics

Statistical analysis was performed using *R* 4.4.3. Data were presented as median with IQR, mean with standard deviation (SD), as absolute (*n*) or relative frequencies (%). To assess the discriminatory power of candidate marker genes, we performed receiver operating characteristic (ROC) analysis. For each marker gene, we compared normalised expression values in the target cell type versus all other cell types, treating expression as a continuous predictor. The area under the ROC curve (AUC) was calculated for each gene, along with the optimal threshold based on Youden’s index. Sensitivity and specificity at the optimal threshold, as well as 95% confidence intervals for AUC, sensitivity, and specificity, were determined using 1000 bootstrap replicates. Differences between two groups were assessed using either Student’s t-test or the Mann–Whitney U test, depending on data distribution. For comparisons involving more than two groups, one-way ANOVA or the Kruskal–Wallis test was applied, as appropriate. *p* ≥ 0.05 was classified as not significant, *p* < 0.05 as significant (∗), *p* < 0.01 (∗∗), *p* < 0.001 (∗∗∗), *p* < 0.0001 (∗∗∗∗).

### Role of funders

The funders had no role in the study design, data collection and analysis, decision to publish, interpretation, or writing of report.

## Results

### An integrated atlas of human skeletal muscle

**Fig. 1:**
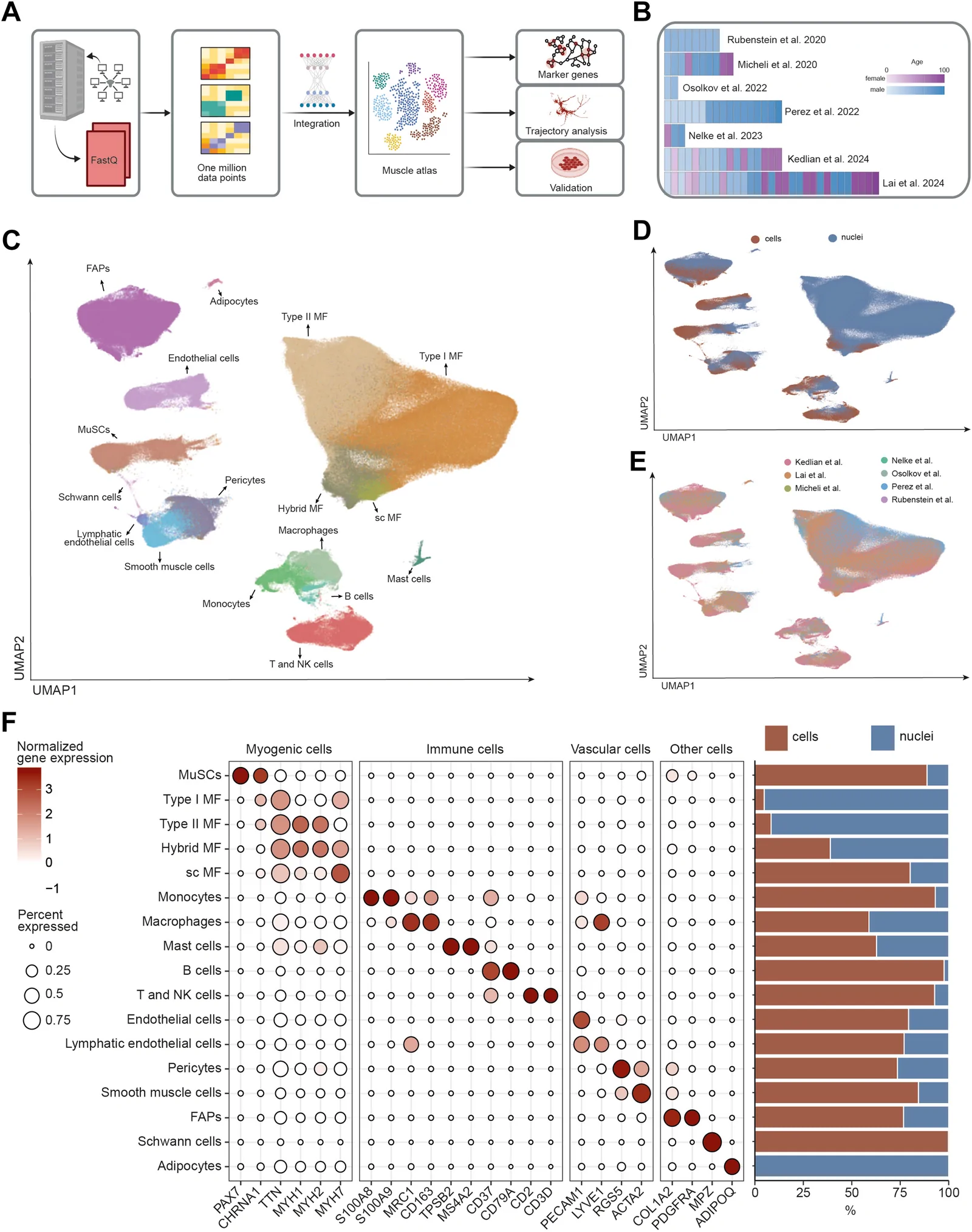
**An integrated atlas of skeletal muscle.** All panels **(A–F)** are based on the complete study cohort of 88 samples. **(A)** Overview of the study design and cohort. **(B)** Overview of the included patients and the number of patients per study. The age and sex are indicated. **(C)** UMAP of the full integrated dataset. The datasets were integrated with Harmony. Clusters were identified by shared nearest neighbour modularity optimisation. After quality control, 122,194 cells and 630,843 nuclei were included in the final dataset. **(D + E)** UMAP with the origin of each cell or nuclei overlaid split by modality or study of origin. **(F)** Dot plot indicating the normalised expression of marker genes and the percentage of cells per cell type expressing these genes. On the right, a bar plot indicates the percentage of cells or nuclei constituting each cluster. **Abbreviations:** FAP, fibro-adipogenic progenitor; MF, myofiber; MuSC, muscle stem cell; UMAP, Uniform manifold approximation and projection; sc, single cell; sn, single nuclei.

To construct a reference atlas, we systematically reviewed the literature to identify publicly available datasets utilising scRNA-seq or snRNA-seq to analyse human skeletal muscle samples (Fig. 1A). Seven studies met our inclusion criteria (Supplementary Table S1). We extracted all data from these studies, cumulating in a combined number of approximately one million cells and nuclei derived from 88 individuals (Fig. 1B). After applying quality control filtering across all datasets, we retained 122,194 cells and 630,843 nuclei for downstream analyses.

To project the scRNA-seq and snRNA-seq datasets into a shared space, we performed a joint integration of all samples. We tested and compared three integration strategies: Harmony, canonical correlation analysis (CCA), and reciprocal principal component analysis (RPCA). Among the tested methods, Harmony most effectively balanced batch correction with biological signal preservation (Fig. 1C–E, Supplementary Fig. S2a and B and Table S2). This integration approach resulted in 17 distinct cell clusters corresponding to previously described cellular states in skeletal muscle (Fig. 1F). Consistent with previous studies, mononuclear cell populations were composed of both scRNA-seq and snRNA-seq data, whereas myofibers (MF) were primarily derived from snRNA-seq as expected due to the inability to process entire MF with scRNA-seq. A small population of MF detected by scRNA-seq likely represents cell fragments generated during tissue dissociation.

For cell annotations, we first employed known marker genes from the literature to manually label each cell type. To evaluate and benchmark against recently developed automated annotation tools, we applied the CHETAH, SingleR, and Azimuth pipelines. We note that this comparison was performed against broad reference frameworks, which are not optimised for skeletal muscle and do not comprehensively represent specialised muscle-resident identities. Accordingly, CHETAH was unable to assign the majority of cells/nuclei in our atlas to known cell types (Supplementary Fig. S3A). SingleR produced spurious annotations, labelling cell types typically not present in muscle tissue, such as osteoblasts, and identifying myogenic cells, including MF, as astrocytes (Supplementary Fig. S3B). Azimuth lacks a skeletal muscle reference dataset, rendering it unsuitable for accurate annotation. Collectively, these findings underscore the need for tissue-specific reference datasets to harmonise studies on muscle transcriptomics.

Having established an integrated atlas, we next leveraged this reference to derive tissue- and modality-aware marker transcript panels.

### Marker genes in non-myogenic cell types

Cell type annotation in single-cell studies frequently relies on canonical marker genes used to define specific cell lineages, often established in disparate biological contexts or model organisms. While these canonical markers have proven invaluable, they may lack the specificity or sensitivity necessary for accurate annotation when applied to new tissue- and technology-specific settings.

**Fig. 2:**
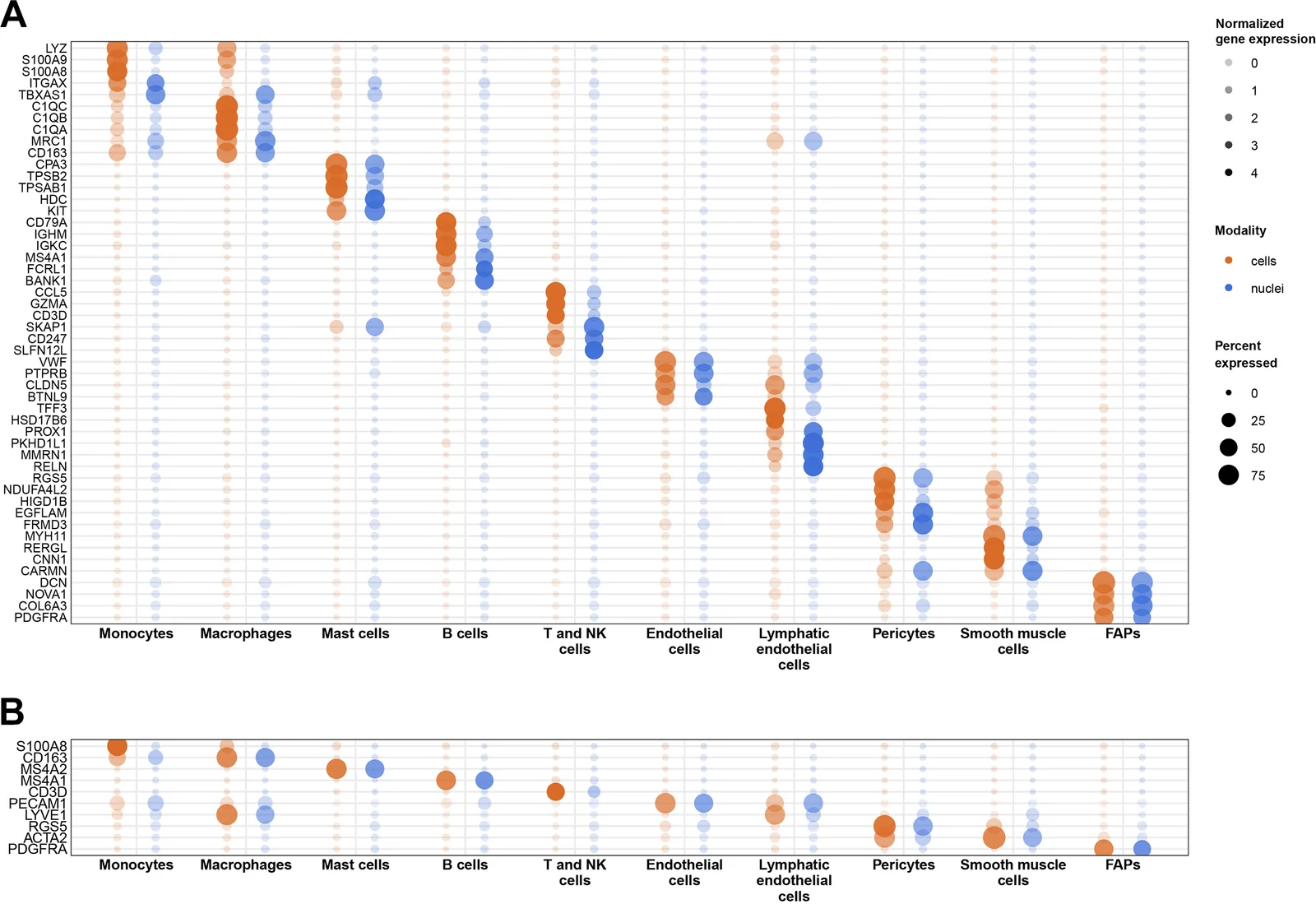
**Unbiased and canonical marker genes (A) Unbiased marker genes for non-myogenic skeletal muscle cell populations shown as a split dot plot comparing single-cell and single-nucleus modalities.** The analysis included the complete study cohort (n = 88 samples; full dataset: 122,194 cells and 630,843 nuclei). Marker genes were identified separately for cells and nuclei using a one-versus-rest design for each annotated cell type. The top positive markers per cell type and modality were selected based on fold-change, expression specificity, and adjusted *p*-value. The displayed gene list represents the union of selected markers from both modalities. **(B)** Canonical marker genes visualised with the same split-dot design. The analysis included the complete study cohort (n = 88 samples). Dot size indicates the percentage of cells or nuclei expressing the gene, and colour indicates scaled normalised expression. Orange denotes single-cell data and blue denotes single-nucleus data. Adipocytes, and Schwann cells were excluded to retain cell populations represented across both modalities and their top marker genes are displayed in Supplementary File S1. **Abbreviations:** FAP, fibro-adipogenic progenitor; MF, myofiber; MuSC, muscle stem cell; UMAP, Uniform manifold approximation and projection; sc, single cell; sn, single nuclei.

To overcome these limitations and define muscle-specific, modality-aware marker transcript lists, we performed an unbiased differential expression analysis across all annotated cell types in our atlas. To account for the impact of sequencing technology on marker detection, we performed separate analyses for scRNA-seq and snRNA-seq data (Fig. 2A) and compared our analysis to a list of canonical marker genes (Fig. 2B). We also provide the full list of marker genes (Supplementary File S1). Notably, we analysed myogenic and non-myogenic cells separately, recognising that the myogenic cell lineage constitutes a developmental continuum that is captured only by snRNA-seq due to the multi-nucleated nature of later stage myogenic cells.

The analysis of non-myogenic cells underscored the influence of sequencing modality on marker gene detection and, by extension, on cell type annotation. Specifically, we corroborate that several canonical marker genes detected by snRNA-seq exhibit limited resolution for mononuclear populations, particularly immune and vascular cell types, likely due to reduced cytoplasmic transcript capture. For instance, the canonical monocyte (and neutrophil) marker *S100A8* is detected in scRNA-seq but not in snRNA-seq data. Similarly, marker combinations such as *RGS5* and *ACTA2*, that allow to distinguish pericytes and smooth muscle cells in scRNA-seq,^1^^,^^2^ provide less discriminative power in our snRNA-seq dataset. In the context of muscle, our unbiased approach nominates new marker genes for specific cell types, e.g., NOVA Alternative Splicing Regulator 1 (*NOVA1*) for fibro-adipogenic progenitors (FAPs). Our findings underscore the necessity of considering both technical and biological context in the interpretation of single-cell data and advocate for the use of tissue- and modality-aware marker panels in muscle research.

To address this need, we developed an interactive, web-based cell-type prediction tool that rapidly and accurately annotates muscle cell populations without requiring any coding expertise. The prediction tool and full reference atlas are publicly accessible for cross-reference (https://annomuscle.shinyapps.io/celltype_shinyapp/). The tool enables users to upload a ranked list of marker genes derived from a cell type or dataset of interest. It then performs a weighted cross-referencing analysis against our atlas-derived marker gene database, providing a prediction of the most likely cell type. It should be noted that we focused on annotating global cell types rather than subdividing clusters into putative cellular subtypes, as the latter are highly context-dependent and require careful interpretation within the framework of individual experimental designs.

We benchmarked our tool on an independent snRNA-seq dataset profiling inflamed skeletal muscle to evaluate its performance under disease conditions.^3^ The ranked marker gene list reported in the original study was used as input and scored against all our reference cell types. The analysis showed concordance between the external annotations and the atlas-derived predictions across cell types (Supplementary Fig. S4A and B). Notably, damaged and reactive MF populations described in the external dataset were not reliably assigned to matching identities, likely because these states emerge in diseased tissue and are not represented in our healthy reference atlas. This observation highlights the need to interpret marker-based annotation in relation to tissue context and disease state. Overall, these results support the use of our marker database as an independent reference for cell-type annotation in skeletal muscle. By contrast, automated annotation tools were unable to reliably match several biologically relevant populations, such as, for example, MuSCs or type I MF (Supplementary Fig. S4C and D). Together, these findings suggest that a tissue-specific reference framework can overcome important limitations of broadly applied annotation tools, particularly for specialised skeletal muscle cell identities.

### Validation of novel FAP lineage markers

As our unbiased profiling nominated novel marker genes, we next sought to validate these candidates in a relevant cell population. FAPs are muscle-resident, mesenchymal cells and the lineage precursor to fibroblasts/myofibroblasts, adipocytes or osteogenic cells.

**Fig. 3:**
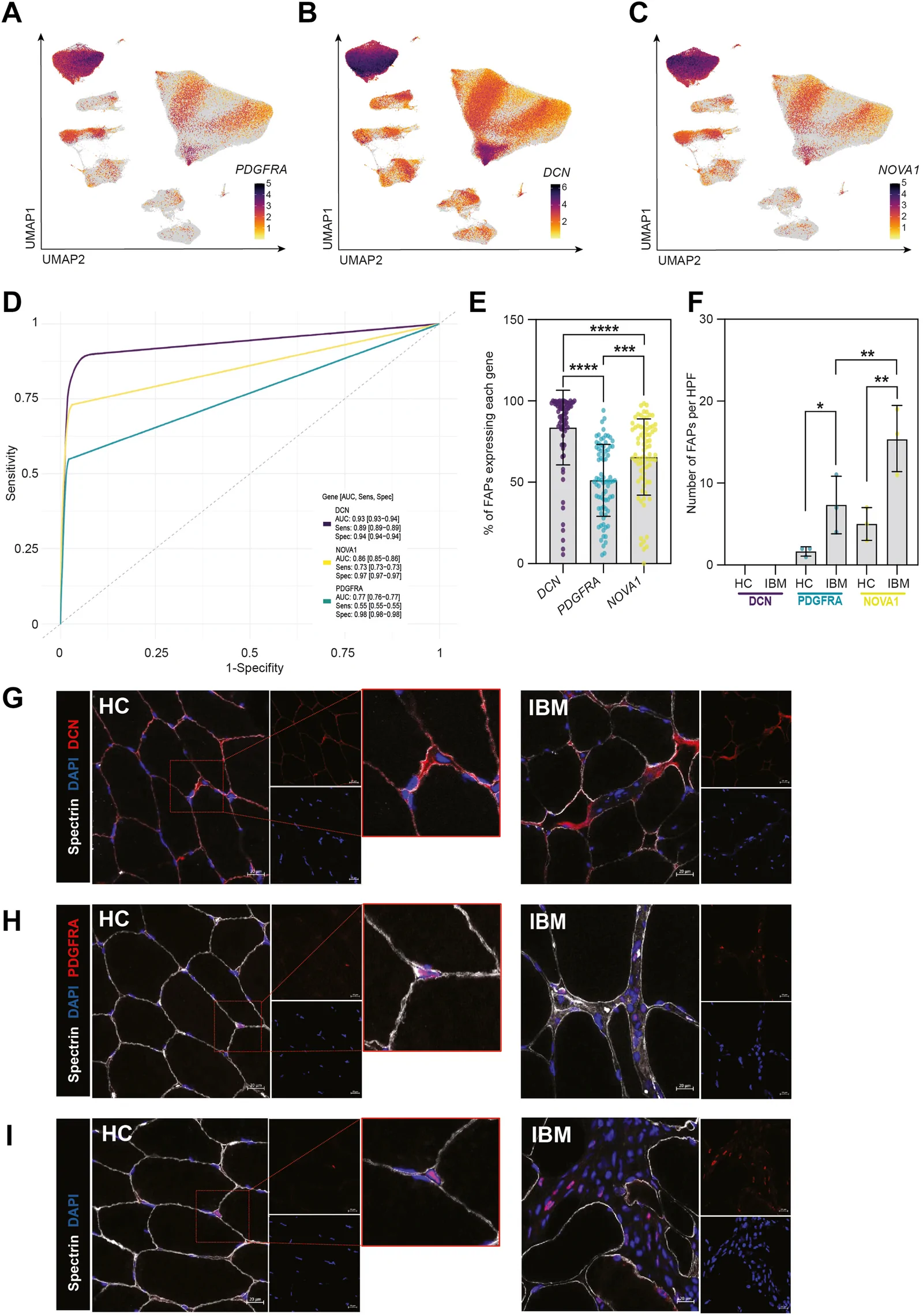
***NOVA1* is a novel marker for the FAP lineage. (A–C)** UMAPs of the full dataset with the normalised expression of the indicated genes overlaid (A: *PDGFRA*, B: *DCN*, C: *NOVA1*). The analysis included the complete study cohort (n = 88 samples). **(D)** ROC curve for the indicated genes for predicting if a cell belongs to the FAP cluster (n = 88 samples). The area under the curve (AUC), sensitivity (Sens), specificity (Spec), and the 95% confidence interval are indicated. **(E)** Percentage of FAPs per sample expressing each indicated marker gene in the transcriptomic atlas (n = 88 samples). Each dot represents one sample contributing to the dataset. Groups were compared by ANOVA, followed by FDR correction for multiple testing. **(F)** Absolute number of FAPs positive for the indicated marker per high-power field (HPF) in muscle biopsies from healthy controls (HC, n = 3) and patients with inclusion body myositis (IBM, n = 3), as assessed by immunofluorescence. Groups were compared by two-sided Student’s t-test, followed by FDR correction for multiple testing. Only perimysial cells were counted. No FAPs could be clearly identified by DCN staining **(G–I)** Representative immunofluorescence images of each marker in HC and IBM. One representative sample is shown per marker and group. The scale bar is 20 μm ∗*p* ≤ 0.05, ∗∗*p* < 0.01, ∗∗∗*p* < 0.001, ∗∗∗∗*p* < 0.0001. **Abbreviations:** FAP, fibro-adipogenic progenitor; UMAP, Uniform manifold approximation and projection.

From our marker lists, we selected highly specific genes for the FAP lineage—decorin (*DCN*), Platelet-Derived Growth Factor Receptor A (*PDGFRA*), and *NOVA1*—for further analysis. *DCN* and *NOVA1* were chosen as highly ranked genes selected from an unbiased approach, while *PDGFRA* is an established marker gene. An overview of the top 10 FAP markers is given in Supplementary Fig. S5A and B. *DCN*, which encodes a proteoglycan present in connective tissue,^22^ was highly expressed in FAPs. However, lower expression levels were observed in hybrid MFs and MF fragments (Fig. 3A). *PDGFRA* was proposed as a lineage marker for FAPs, indicating a FAP subtype favouring adipogenesis.^23^ This gene was also detected as a FAP marker by our unbiased approach (Fig. 3B). Finally, we also identified *NOVA1* as a candidate marker gene for FAPs. Surprisingly, although considered as a neuron-specific RNA-binding protein,^24^ *NOVA1* was also highly and selectively expressed in FAPs (Fig. 3C), suggesting a previously unrecognised role in muscle-resident cells. To evaluate the utility of these candidate marker genes for identifying FAPs, we assessed their discriminatory performance using receiver operating characteristic (ROC) analysis. *DCN* had the highest AUC with 0.93 (95% CI: 0.93 to 0.94, sensitivity: 89% (89–89%), specificity: 94% (94–94%)) for distinguishing FAPs from all other cell types (Fig. 3D). *NOVA1* showed a comparable AUC of 0.86 (95% CI: 0.85 to 0.86, sensitivity: 73% (73–73%), specificity: 97% (97–97%), while *PDGFRA* exhibited a lower overall accuracy (AUC: 0.77, 95% CI 0.76 to 0.77, sensitivity: 55% (55–55%), specificity: 98% (98–98%)). To further examine the robustness of these markers, we quantified the proportion of FAPs expressing each marker gene per individual sample. Notably, we observed substantial inter-sample variability (Fig. 3E). The median percentage of FAPs expressing *DCN* was 92.4% (IQR: 15.2%), compared to 66.9% (IQR: 30.4%) for *NOVA1* and 50.8% (IQR: 37.1%) for *PDGFRA*. These data highlight that, while *DCN* demonstrates robust and consistent expression across samples, the performance of other markers varies considerably with the biological contexts.

To evaluate the robustness of our candidate FAP markers across experimental modalities, we performed immunofluorescence (IF) staining for each marker in muscle biopsies from both healthy controls (HCs) and patients with inclusion body myositis (IBM). This approach allowed us to assess whether markers identified by unbiased transcriptomics are faithfully represented at the protein level within tissue and to determine their utility under both physiological and disease conditions. In this context, IBM is characterised by substantial expansion and activation of the FAP lineage, promoting fibrosis and tissue remodelling. Our IF analyses demonstrated that DCN effectively labelled the perimysial space, with a stronger signal observed in IBM compared to HC samples, in line with an expansion of this cell type and its known role as an extracellular matrix protein (Fig. 3G). However, DCN did not clearly indicate specific cells due to a lack of cellular staining, rendering it unsuitable for analysis by IF. In contrast, PDGFRA demonstrated highly specific staining of individual perimysial cells (Fig. 3H), although fewer cells were detected than for DCN or NOVA1, consistent with our transcriptomic findings. NOVA1 provided a trade-off between the former two markers, selectively labelling individual cells and identifying a greater number of FAPs than PDGFRA (Fig. 3I). To validate *NOVA1* in an unintegrated reference dataset, we queried the Skeletal Muscle Ageing Atlas.^2^ In the human dataset, *NOVA1* expression was specific in the fibroblast populations, corresponding to the FAP label in our annotation framework (Supplementary Fig. S6A and B). We next examined the mouse dataset from the same study and observed a similar enrichment of *Nova1* in fibroblasts (Supplementary Fig. S6C and D). These findings support *NOVA1* as a marker of the fibroblast/FAP compartment in skeletal muscle and suggest that this expression pattern is conserved across species.

To gain a putative understanding of the biological function of *NOVA1* in FAPs, we treated *NOVA1* as a continuous variable and calculated the co-expression network (Supplementary Fig. S7A and File S2A–D). The most strongly correlated genes included splicing-associated factors, e.g., *SMAD3*,^25^ and transcription factors such as *CREB5*^26^ or *TNXB*.^27^ Gene-set enrichment of genes correlating with *NOVA1* expression revealed signals for cytoplasmic translation, rRNA processing, and mRNA splicing (Supplementary Fig. S7B), in line with *NOVA1*’s known role as a splicing regulator.^28^ These data suggest that NOVA1 might be a part of a regulatory gene module in FAPs, linking splicing control to broader cellular programs that may influence FAP state and function.

Collectively, these results highlight the value of unbiased transcriptomic approaches for the identification of novel, context-specific cellular markers, such as *NOVA1*, in muscle. Overall, while *DCN* emerged as the strongest transcript-level marker of the FAP lineage, NOVA1 proved particularly useful for IF detection under our experimental conditions, whereas DCN primarily highlighted the extracellular matrix. These findings should be interpreted cautiously, as IF detectability may be influenced by protein localisation and antibody performance in addition to transcript abundance.

### The myogenic lineage in muscle transcriptomics

Having defined tissue- and modality-aware marker panels, we next focused on the myogenic compartment. For this purpose, we extracted all myogenic cells from the full atlas and restricted subsequent analyses to snRNA-seq data, given that MF are only captured by this modality given their size.

**Fig. 4:**
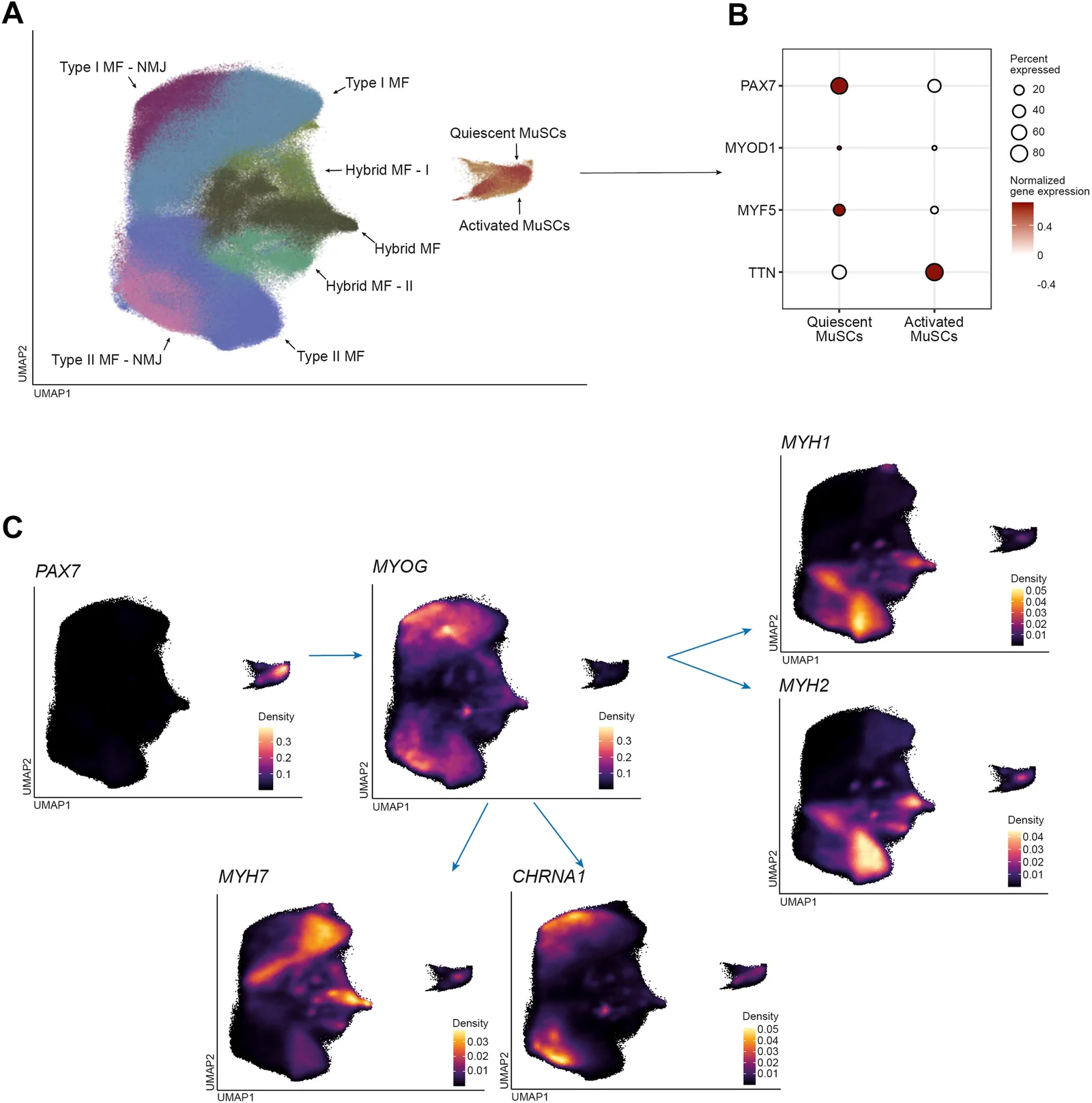
**All panels (A–C) are based on the complete study cohort of 88 samples. (A)** UMAP of the myogenic nuclei. We retained only single nuclei classified as myofiber (MF) for downstream analysis. The datasets were integrated with Harmony. Clusters were identified by shared nearest neighbour modularity optimisation. The UMAP indicates each MF population. **(B)** Dot plot showing the marker genes for the MuSC cell type. Dot plot indicated normalised gene expression and percent expressed. *PAX7*, *MYOD1* and *MYF5* mark a quiescent MuSC phenotype, while engagement of *TTN* indicates activation. **(C)** Nebulosa plots indicating the cell density overlaid on the UMAP for the indicated genes. Gene-weighted kernel density estimation as used to visualise cells expressing a given gene. Blue arrows demonstrate the biological sequence of MF differentiation into either MF type or into neuromuscular junctions. *PAX7* marks MuSCs, while *MYOG* indicates early differentiating MFs. Type I MF express *MYH1* and *MYH2*, while type II MF express *MYH7*. *CHRNA1* indicates MFs contributing to NMJ formation. **Abbreviations:** MuSC, muscle stem cell; NMJ, neuromuscular junction; UMAP, Uniform manifold approximation and projection.

We then subclustered this compartment to resolve its transcriptional heterogeneity (Fig. 4A). This approach identified nine distinct cellular clusters, which we annotated based on established markers and the known trajectory of myogenic differentiation. In line with biological expectation, the resulting clusters encompassed a main MF cluster and a MuSC population distinguished by *PAX7* expression (Fig. 4B and C), with MF referring to myofiber-associated nuclei captured by snRNA-seq rather than intact myofibers. The MuSC population could be further resolved into a quiescent MuSC subset characterised by high *PAX7* levels, as well as an activated or early differentiating MuSC cluster defined by downregulation of *PAX7* and engagement of *TTN*. Although MYOD1 is expected to mark MuSCs undergoing commitment to differentiation, it was not reliably detected, likely reflecting the lower sensitivity of snRNA-seq for certain transcripts or different behaviour between the transcript and protein levels.

To identify specific marker genes that indicate all nuclei contributing to either the type I or type II MF lineage, we performed differential analysis comparing the two trajectories. We found that the skeletal muscle troponin genes *TNNT1* and *TNNT3* were consistently expressed across their respective lineages: *TNNT1* marked all type I MF and their precursors, while *TNNT3* labelled the entire type II MF trajectory (Supplementary Fig. S8A and B). In contrast to myosin heavy chains (MYH), which are restricted to adult MF subtypes (Supplementary Fig. S8C and D), troponin gene expression thus provides a continuous marker for tracking lineage progression, including both differentiated MFs and those in transition.

Analysis of differentiated MF populations further revealed that snRNA-seq faithfully captures the known biological sequence of myogenic differentiation. Early-stage myofibers exhibited a hybrid MYH expression pattern—including *MYH1*, *MYH2*, and *MYH7*—consistent with a transitional developmental state. As MF matured, they progressed toward either a type I or type II MF identity, transiently expressing *MYOG* during differentiation before ultimately adopting an adult-specific MYH isoform profile. Within the differentiated compartment, we also resolved neuromuscular junction (NMJ)-associated MF populations along both type I and type II trajectories, distinguished by expression of the acetylcholine receptor (*CHRNA1*) and neuromuscular junction markers such as *MUSK*.

Collectively, these findings underscore snRNA-seq as a modelling tool for myogenic differentiation, with our dataset providing a reference framework to guide lineage analysis in future studies.

### Developmental trajectories in type I and type II myofibers

**Fig. 5:**
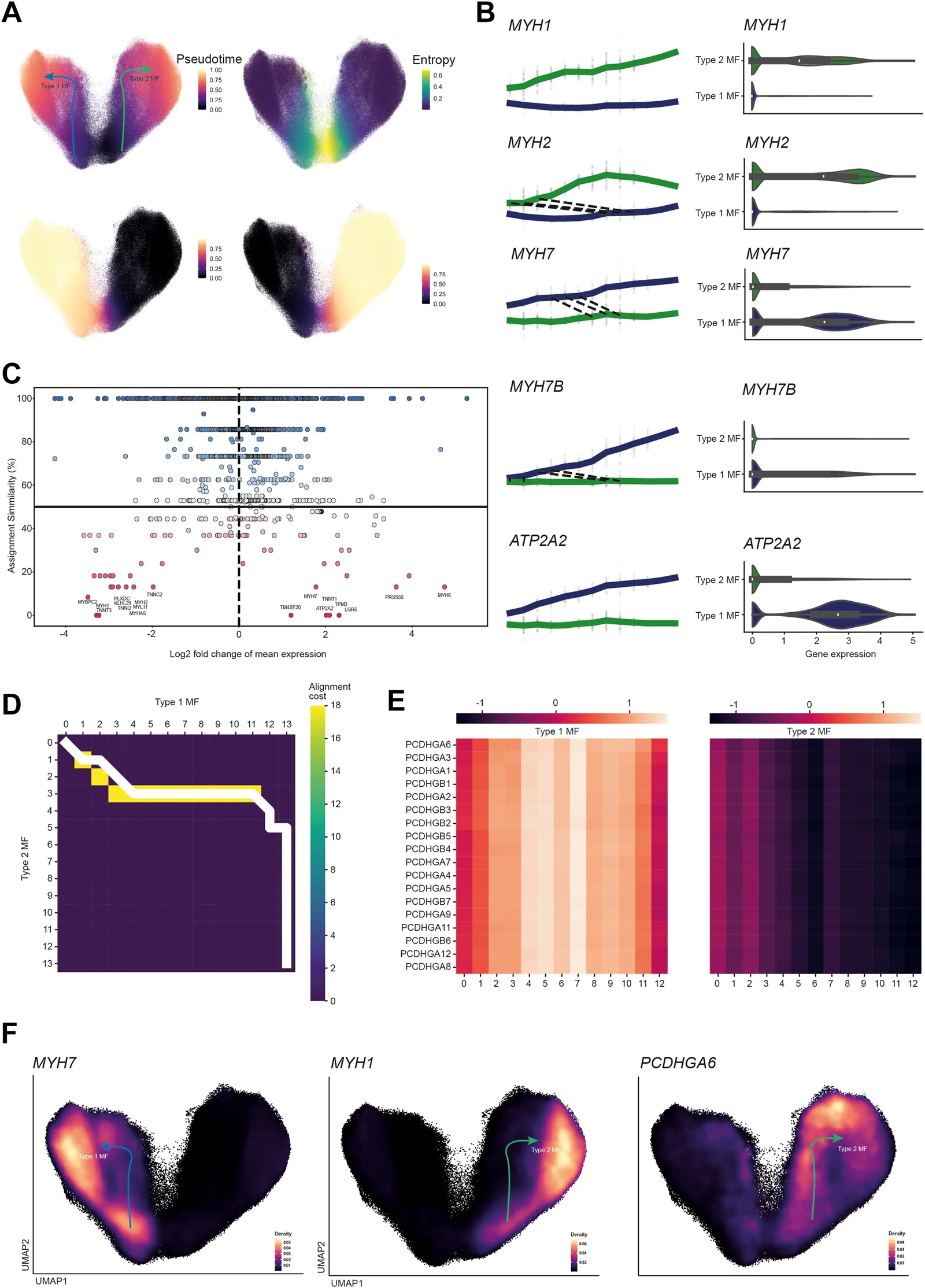
**All panels (A–F) are based on the complete study cohort of 88 samples. (A)** Pseudotime trajectories computed with Palantir align cells along differentiation trajectories. The pseudotime values for each nuclei were normalised. The type I and type II MF trajectory are indicated in blue or green, respectively. The lower two plots indicate the pseudotime for the type I MF trajectory (left) or type II MF trajectory (right). **(B)** Gene-level trajectory alignment using the Genes2Genes framework and highly variable genes as input. Alignment was inferred to match gene-expression trajectories across pseudotime for both MF types. The nuclei were placed into bins based on their pseudotime. The expression for each bin is indicated for type 1 MF (blue) and type 2 MF (green). Violin plots show the overall gene expression for each MF type. **(C)** Scatter plot showing the log2 fold change of the mean expression comparing type 1 (right) and type 2 (left) MF and the assignment similarity. The assignment similarity quantifies the degree to which the pseudotime trends between the two MF trajectories are similar. **(D)** Alignment cost heatmap between type 1 and type 2 MFs. The alignment cost is computed as the difference between the two gene-expression profiles over time, with higher values indicating greater divergence. The cost accounts for both matches and mismatches in the alignment. **(E)** Pseudotime heatmap for type 1 and type 2 MF. The protocadherin gamma (*PCDHG*) gene cluster is indicated with the normalised gene expression for each pseudotime bin. **(F)** Nebulosa plots demonstrating the density of nuclei expressing the indicating genes. *PCDHGA6* was chosen at random to demonstrate expression of a given *PCDHG* gene. **Abbreviations:** MF, myofiber; UMAP, Uniform manifold approximation and projection.

To test whether our atlas can reconstruct the developmental logic of human myogenesis, we modelled MF differentiation using pseudotime analysis. The myogenic lineage encompasses the progression from early MuSCs to fully differentiated MFs with specialised contractile and metabolic properties. For pseudotime inference, we interpreted nuclei expressing mature adult MF genes (*MYH1*, *MYH2* and *MYH7*) as terminal differentiated states; this represents a biologically informed assumption used to orient the inferred continuum rather than a direct measurement of time. By ordering all MF nuclei along pseudotime trajectories, we observed a clear bifurcation that separated nuclei committing to either a type I or type II MF identity (Fig. 5A). As expected, the progression along each branch was marked by a decline in transcriptional entropy and a stepwise accumulation of adult MYH isoforms, mirroring the sequence of MF maturation.

With this model, we next asked whether these developmental trajectories would recapitulate established gene expression patterns and potentially reveal novel regulators of myogenesis. To compare gene dynamics between lineages, we applied the Genes2Genes framework, which enables non-linear alignment of trajectories and the identification of gene expression mismatches between the type I and type II branches. This approach captured the sequential upregulation of established lineage-specific genes, including *MYH1* and *MYH2* in the type II trajectory, and *MYH7* and *MYH7B* in the type I trajectory (Fig. 5B, Supplementary File S3). Our analysis also faithfully reflected MF type-specific genes, such as the gradual increase in *ATP2A2* (encoding sarcoendoplasmic reticulum Ca2+-ATPase 2 (SERCA2)) in type I MF (Fig. 5C).^18^

To determine whether trajectory analysis could also uncover previously unrecognised lineage-specific gene programs, we clustered genes by their pseudotemporal expression patterns. We ranked modules by their divergence between the two lineages. Strikingly, this revealed a gene cluster selectively enriched in developing type I MF, comprised entirely of protocadherin (PCDH) genes from the gamma subfamilies A and B (Fig. 5D). These genes displayed expression in early MF type I differentiation, diminishing as MF reached terminal maturation—a pattern absent from the type II lineage (Fig. 5E and F, Supplementary Fig. S9). As the overall *PCDH* expression in mature MF was comparably low, this trajectory-specific peak is expected to be less visible in static, cross-sectional analyses, given a lower magnitude of change. Indeed, to cross-validate this finding in an independent dataset, we queried a recent study analysing approximately 1000 individual MF for type-specific gene and protein patterns.^18^ We extracted the transcriptomic data for all detected *PCDH* genes and observed a similar pattern of modest upregulation (*PCDH7*, *PCDH9* and *PCDH17*) in type I compared to type II MF as in our trajectory analysis (Supplementary Fig. S10A). Concurrently, we also re-analysed the proteomic profiling of the external dataset and queried all proteins for PCDH. Indeed, PCDH1 was also increased on the protein level in type I compared to type II MF (Supplementary Fig. S10B). Notably, no genes of the gamma subfamily were detected in the external dataset, hindering direct validation of the gene cluster identified in our study.

The transient expression of protocadherins during type I MF differentiation suggests a conserved regulatory mechanism between muscle and neural tissues, underscoring the utility of trajectory-based modelling in detecting novel gene programs implicated in myogenesis.

## Discussion

In this study, we present a unified reference atlas of human skeletal muscle, harmonising currently available single-cell and single-nucleus data. This resource establishes a framework for muscle research, readily accessible through an interactive annotation tool, and offers a comprehensive overview of modality- and tissue-specific marker genes.

Our approach is situated within the movement towards large-scale atlas initiatives, such as the Human Cell Atlas and Tabula Sapiens, which have transformed our ability to interpret single-cell data across tissues.^7^^,^^8^^,^^10^ However, skeletal muscle presents unique technical and biological challenges. The MF, the primary functional unit of muscle, is a large multinucleated syncytium that is poorly captured by scRNA-seq alone. Earlier atlases, including a pioneering study analysing approximately 22,000 cells from 10 donors,^29^ highlighted the heterogeneity of muscle-resident cells, but were limited to non-myogenic populations. By integrating both scRNA-seq and snRNA-seq into a single joint atlas, we overcome these limitations, enabling profiling of all muscle cell types—including MF and their intermediate differentiation stages within the myogenic lineage. While this dual-modality integration maximises cellular representation, it also exposes modality-specific biases, such as reduced detection of cytosolic transcripts in snRNA-seq.^1^^,^^2^^,^^30^^,^^31^ To address this, we provide modality-aware marker lists that enable cell-type annotation across platforms.

We also expand on recent efforts to map MF heterogeneity at the transcriptomic and proteomic levels. Building on prior findings that troponin genes *TNNT1* and *TNNT3* serve as markers for type I and type II MF,^18^ the application of single nuclei resolution reveals that these genes are expressed not only in mature MF but also in early differentiating stages. This feature makes them valuable tools for tracking fibre type commitment during muscle development, extending beyond the restricted expression of *MYH* isoforms to terminally differentiated MF.

Our analysis also uncovers unexpected molecular parallels between muscle and the nervous system. We validate *NOVA1*—a gene previously considered neuron-specific^24^^,^^28^^,^^32^—as a highly specific marker for FAPs in muscle, underscoring the convergence of regulatory programs between tissues. Indeed, the identification and annotation of FAPs has remained challenging across studies.^1^^,^^2^^,^^31^^,^^33^^,^^34^ Mechanistically, *NOVA1* is known to control alternative splicing and to regulate transcript stability through binding to nonsense-mediated decay exons.^35^ Recently, *NOVA1* has been shown to modulate transforming growth factor beta (TGF-β) signalling,^36^ providing a potential functional link to muscle, as TGF-β is a central regulator of FAP activation and differentiation.^34^ We therefore hypothesise that *NOVA1* acts as a splicing regulator in FAPs, tuning their response to TGF-β cues during muscle regeneration and fibrosis. Furthermore, the reconstruction of myogenic differentiation reveals a transient and selective activation of protocadherin gamma genes in the type I MF lineage. This finding underscores the value of pseudotime-resolved trajectory analysis in capturing stage-specific differentiation programs.^15^^,^^37^ Interestingly, studies in mice demonstrate that motor innervation can repress certain developmental genes in MF,^38^ and that fibre-type specific gene programs rely on neural input. For instance, a loss of Ca^2+^ influx impairs MF maturation by failing to induce the transcription factor Maf.^38^ The transient expression of protocadherins in developing type I MF may reflect a similar regulatory logic. Protocadherins may be required for axonal guidance and the MF-specific establishment of signalling via the neuromuscular junction. Supporting this, a previous study detected protocadherin gamma expression in skeletal muscle and observed an association with ageing.^39^ As aged muscle displays signs of denervation,^1^^,^^2^ increased protocadherin expression may represent a compensatory mechanism that promotes neuromuscular synaptic signalling.

Several limitations of this study should be acknowledged. First, this atlas is restricted to healthy adult human skeletal muscle and therefore does not capture the substantial remodelling that occurs in muscle conditions such as disease or ageing. As a result, the present resource is intended as a baseline reference framework, rather than a comprehensive catalogue of all muscle cell states encountered across physiological and pathological contexts. Future integration of diseased and perturbed muscle will be essential to define context-dependent cell states and shifts in cellular composition relative to this healthy reference.

Second, the atlas is assembled from previously published datasets, which necessarily introduces heterogeneity in sample handling, nuclei or cell isolation, sequencing chemistry, and data availability. Although we benchmarked several integration methods, technical variation cannot be fully eliminated. Related to this, the joint use of scRNA-seq and snRNA-seq increases the breadth of cellular representation, particularly by enabling capture of multinucleated MF, but also introduces modality-specific biases in transcript detection. We addressed this by generating modality-aware marker panels; nevertheless, users should interpret marker performance in the context of the underlying sequencing platform. Third, our study remains largely descriptive. The identification of candidate regulators and lineage-associated programs, including those involving *NOVA1* and protocadherin family members, provides hypotheses for future work, but does not establish functional causality. Their biological roles will require targeted perturbation and mechanistic validation. Finally, the present atlas lacks spatial information. Because skeletal muscle function depends strongly on tissue architecture and local cellular interactions, integration with spatial transcriptomics and imaging-based approaches will be important to resolve anatomical niche structure, cell–cell interactions, and microenvironmental organisation in future studies. The weak correlation between *PCDHG* genes and *MYH7* at the level of individual nuclei limits the evidence for direct co-expression in type I MFs. This discrepancy may reflect distinct temporal expression windows, with *PCDHG* expression occurring earlier along the inferred type I differentiation trajectory and *MYH7* predominantly marking later, more differentiated MF nuclei; accordingly, the *PCDHG* finding should be interpreted as a trajectory-associated program rather than evidence of sustained co-expression in mature *MYH7*-positive MFs.

In summary, our atlas provides a starting point for a reference framework in muscle biology. We anticipate that this resource will resolve inconsistencies in cell-type annotation, enable reproducible cell identification, and serve as a baseline for future studies exploring muscle development, regeneration, disease, and intervention.

## Contributors

CN and TR designed the study, supervised the analysis, and wrote the manuscript. CN and FB performed the literature review and the analysis. AKG, CBS, PQ, KK, GMzH, BS performed experiments and revised the manuscript. JHWD, SGM, FK, and TR provided funding, contributed substantially to the interpretation of the work and revised the manuscript. CBS performed experiments and revised the manuscript substantially. CN, FK and TR accessed and verified the underlying data. All authors read and approved the final version of the manuscript and are accountable for all aspects of the work.

## Data sharing statement

The complete dataset is publicly available at https://annomuscle.shinyapps.io/celltype_shinyapp. This includes the full transcriptomic dataset produced in this study. The raw data, including Seurat and H5AD objects, accompanying metadata and documentation, the underlying code, and marker tables for each cell type are available at https://doi.org/10.5281/zenodo.21681522. The data is available without an end date.

## Declaration of interests

All authors completed the ICMJE disclosure forms. CN declares honoraria for lectures from ArgenX and Alexion. TR declares consulting fees and lecture honoraria from ArgenX, Alexion, UCB, JNJ, Merck, Novartis, Sanofi, and Amgen, and participation on a DSMB for ArgenX/efgartigimod. GMzH declares consulting fees from ArgenX, JnJ, and Sanofi; lecture honoraria from ArgenX, Alexion, JnJ, Sanofi, and Amgen; and travel support from JnJ. SGM declares consulting fees and/or lecture honoraria from Academy 2, ArgenX, Alexion, Almirall, Amicus Therapeutics Germany, Bayer Health Care, Biogen, BioNTech, BMS, Celgene, Datamed, Demecan, Desitin, Diamed, Diaplan, DIU Dresden, DPmed, Gen Medicine and Healthcare Products, Genzyme, Hexal, IGES, Impulze, Janssen-Cilag, KW Medipoint, MedDay Pharmaceuticals, Medudy, Merck Serono, MICE, Mylan, Neuraxpharm, Neuropoint, Novartis, Novo Nordisk, ONO Pharma, Oxford PharmaGenesis, QuintilesIMS, Roche, Sanofi-Aventis, Springer Medizin Verlag, STADA, Chugai Pharma, Teva, UCB, Viatris, Wings for Life International, and Xcenda. PQ declares lecture honoraria and travel support from Merck, Novartis, and ArgenX. KK declares lecture honoraria from Sanofi-Aventis. JHWD declares grants or contracts from AbbVie, Agomab, Anamar, ArgenX, ARXX, Boehringer Ingelheim, Candid, Cantargia, Celgene, CSL Behring, Eli Lilly, ExoTherapeutics, GSK, Lassen, Kyverna, Mestag, Novartis, Otsuka, and UCB; consulting fees from AbbVie, Anamar, ARXX, AstraZeneca, Boehringer Ingelheim, Genentech/Roche, GSK, Novartis, Roche, Sulis, Spica, Tyra, UCB, and Xenas; lecture or educational honoraria from AbbVie, Anamar, ARXX/Caluna, AstraZeneca, Boehringer Ingelheim, Eli Lilly, Genentech/Roche, GSK, Merck, Novartis, and UCB; expert testimony payments from AbbVie, Anamar, ARXX/Caluna, AstraZeneca, Bayer Pharma, Boehringer Ingelheim, Cantargia, Genentech, Quell, Kyverna, Novartis, Prolium, Roche, and UCB; travel support from AbbVie and SOBI; stock or stock options from 4D Science and FibroCure; and receipt of materials or services from Boehringer Ingelheim. BS declares grants or contracts from DFG, Horizon 2022 PALADIN, COMPASS, and Entry-DM outside the submitted work; consulting fees from Avidity, Amicus, Alexion, ArgenX, Astellas, Bayer, Denali, Dyne, and Sanofi; lecture honoraria from Amicus, Alexion, ArgenX, Astellas, Dyne, and Sanofi; and participation on advisory boards for Endoced and Arthex. FK declares grant support from DFG outside the submitted work. CBS, FB and AKG declare no competing interests.

## Appendix A Supplementary data

Supplementary File 3

Supplementary File Captions
