# Supplementary material for: An integrated reference atlas of human skeletal muscle
Source: eBioMedicine. 2026 Aug 31;131:106465. doi: 10.1016/j.ebiom.2026.106465 (PMC13562069; doi:10.1016/j.ebiom.2026.106465)
Supplement: Supplementary file 1 [file mmc1.docx]

**An Integrated Reference Atlas of Human Skeletal Muscle**

**Supplementary Figures, Tables and Files**

Supplemental Figure 1: Page 2

Supplemental Table 1: Page 3 to 7

Supplemental Figure 2: Page 8 to 9

Supplemental Table 2: Page 10

Supplemental Figure 3: Page 11

Supplemental File 1: Excel Sheet “Suppl_File_1.xlsx”

Supplemental Figure 4: Page 12

Supplemental Figure 5: Page 13

Supplemental Figure 6: Page 14

Supplemental Figure 7: Page 15

Supplemental File 2: Excel Sheet “Suppl_File_2.xlsx” with A to D

Supplemental Figure 8: Page 16

Supplemental File 3: Excel Sheet “Suppl_File_3.xlsx”

Supplemental Figure 9: Page 17

Supplemental Figure 10: Page 18

**Supplemental Figure 1**

**Supplemental Figure 1: Mitochondrial reads across studies and cell types.** Violin plots indicating the percentage of mitochondrial reads for each study **(A)** and each cell type **(B)**. The box plot indicates the median and error bars indicate the minimum and maximum values.

**Suppl. Table 1**

| **ID** | **SRA Accession** | **Origin** | **Single Nuc/Single Cell** | **Platform** | **Age (years)** | **Sex** | **Preprocessing** | **Muscle** |
| --- | --- | --- | --- | --- | --- | --- | --- | --- |
| GSM5724579/1 | SRR21931094 | Osolkov et al. 2022 | single nuclei | Illumina HiSeq 2000 | 35 | male | CellRanger | Vastus lateralis |
| GSM5724580/2 | SRR21931095 | Osolkov et al. 2022 | single nuclei | Illumina HiSeq 2000 | 45 | male | CellRanger | Vastus lateralis |
| GSM5098737/3 | SRR13759043 | Perez et al. 2022 | single nuclei | Illumina NovaSeq 6000 | 75 | male | CellRanger | Vastus lateralis |
| GSM5098738/4 | SRR13759044 | Perez et al. 2022 | single nuclei | Illumina NovaSeq 6000 | 76 | male | CellRanger | Vastus lateralis |
| GSM5098739/5 | SRR13759045 | Perez et al. 2022 | single nuclei | Illumina NovaSeq 6000 | 75 | male | CellRanger | Vastus lateralis |
| GSM5098740/6 | SRR13759046 | Perez et al. 2022 | single nuclei | Illumina NovaSeq 6000 | 79 | male | CellRanger | Vastus lateralis |
| GSM5098741/7 | SRR13759047 | Perez et al. 2022 | single nuclei | Illumina NovaSeq 6000 | 20 | male | CellRanger | Vastus lateralis |
| GSM5098742/8 | SRR13759048 | Perez et al. 2022 | single nuclei | Illumina NovaSeq 6000 | 82 | male | CellRanger | Vastus lateralis |
| GSM5098743/9 | SRR13759049 | Perez et al. 2022 | single nuclei | Illumina NovaSeq 6000 | 72 | male | CellRanger | Vastus lateralis |
| GSM5098744/10 | SRR13759050 | Perez et al. 2022 | single nuclei | Illumina NovaSeq 6000 | 21 | male | CellRanger | Vastus lateralis |
| GSM5098745/11 | SRR13759051 | Perez et al. 2022 | single nuclei | Illumina NovaSeq 6000 | 70 | male | CellRanger | Vastus lateralis |
| GSM5098746/12 | SRR13759052 | Perez et al. 2022 | single nuclei | Illumina NovaSeq 6000 | 21 | male | CellRanger | Vastus lateralis |
| GSM5098747/13 | SRR13759053 | Perez et al. 2022 | single nuclei | Illumina NovaSeq 6000 | 75 | male | CellRanger | Vastus lateralis |
| GSM5098748/14 | SRR13759054 | Perez et al. 2022 | single nuclei | Illumina NovaSeq 6000 | 72 | male | CellRanger | Vastus lateralis |
| GSM5098749/15 | SRR13759055 | Perez et al. 2022 | single nuclei | Illumina NovaSeq 6000 | 19 | male | CellRanger | Vastus lateralis |
| GSM5098750/16 | SRR13759056 | Perez et al. 2022 | single nuclei | Illumina NovaSeq 6000 | 79 | male | CellRanger | Vastus lateralis |
| GSM5098751/17 | SRR13759057 | Perez et al. 2022 | single nuclei | Illumina NovaSeq 6000 | 22 | male | CellRanger | Vastus lateralis |
| GSM5098752/18 | SRR13759058 | Perez et al. 2022 | single nuclei | Illumina NovaSeq 6000 | 21 | male | CellRanger | Vastus lateralis |
| GSM5098753/19 | SRR13759059 | Perez et al. 2022 | single nuclei | Illumina NovaSeq 6000 | 90 | male | CellRanger | Vastus lateralis |
| GSM3746214/20 | SRR9018527 | Rubenstein et al. 2020 | single cell | Illumina HiSeq 2500 | young | male | CellRanger | Vastus lateralis |
| GSM3746214/21 | SRR9018528 | Rubenstein et al. 2020 | single cell | Illumina HiSeq 2500 | young | male | CellRanger | Vastus lateralis |
| GSM3746215/22 | SRR9018529 | Rubenstein et al. 2020 | single cell | Illumina HiSeq 2500 | young | male | CellRanger | Vastus lateralis |
| GSM3746215/23 | SRR9018530 | Rubenstein et al. 2020 | single cell | Illumina HiSeq 2500 | young | male | CellRanger | Vastus lateralis |
| GSM3746212/24 | SRR9018531 | Rubenstein et al. 2020 | single cell | Illumina HiSeq 2500 | young | male | CellRanger | Vastus lateralis |
| GSM3746212/25 | SRR9018532 | Rubenstein et al. 2020 | single cell | Illumina HiSeq 2500 | young | male | CellRanger | Vastus lateralis |
| GSM3746213/26 | SRR9018533 | Rubenstein et al. 2020 | single cell | Illumina HiSeq 2500 | young | male | CellRanger | Vastus lateralis |
| GSM3746213/27 | SRR9018534 | Rubenstein et al. 2020 | single cell | Illumina HiSeq 2500 | young | male | CellRanger | Vastus lateralis |
| NDC1/ 28 |  | Nelke et al. 2023 | single nuclei | Illumina NovaSeq 6000 | 58 | female | CeleScope | Vastus lateralis |
| NDC2/29 |  | Nelke et al. 2023 | single nuclei | Illumina NovaSeq 6000 | 67 | male | CeleScope | Vastus lateralis |
| NDC3/30 |  | Nelke et al. 2023 | single nuclei | Illumina NovaSeq 6000 | 73 | male | CeleScope | Vastus lateralis |
| 470BR/31 |  | Kedlian et al. 2024 | single cell,single nuclei | Illumina HiSeq 4000/NovaSeq 6000 | 17-20 (young) | male | CellRanger | Intercostal muscle |
| 398B/32 |  | Kedlian et al. 2024 | single nuclei | Illumina HiSeq 4000/NovaSeq 6000 | 20-25 (young) | female | CellRanger | Intercostal muscle |
| 583B/33 |  | Kedlian et al. 2024 | single cell,single nuclei | Illumina HiSeq 4000/NovaSeq 6000 | 25-30 (young) | male | CellRanger | Intercostal muscle |
| 566B/34 |  | Kedlian et al. 2024 | single nuclei | Illumina HiSeq 4000/NovaSeq 6000 | 25-30 (young) | female | CellRanger | Intercostal muscle |
| 411C/35 |  | Kedlian et al. 2024 | single cell,single nuclei | Illumina HiSeq 4000/NovaSeq 6000 | 24-30 (young) | male | CellRanger | Intercostal muscle |
| 464C/36 |  | Kedlian et al. 2024 | single cell,single nuclei | Illumina HiSeq 4000/NovaSeq 6000 | 35-40 (young) | male | CellRanger | Intercostal muscle |
| 529C/37 |  | Kedlian et al. 2024 | single nuclei | Illumina HiSeq 4000/NovaSeq 6000 | 35-40 (young) | male | CellRanger | Intercostal muscle |
| 591C/38 |  | Kedlian et al. 2024 | single cell | Illumina HiSeq 4000/NovaSeq 6000 | 35-40 (young) | male | CellRanger | Intercostal muscle |
| 621B/39 |  | Kedlian et al. 2024 | single cell | Illumina HiSeq 4000/NovaSeq 6000 | 50-55 (old) | male | CellRanger | Intercostal muscle |
| 582C/40 |  | Kedlian et al. 2024 | single cell,single nuclei | Illumina HiSeq 4000/NovaSeq 6000 | 55-60 (old) | female | CellRanger | Intercostal muscle |
| 362C/41 |  | Kedlian et al. 2024 | single cell,single nuclei | Illumina HiSeq 4000/NovaSeq 6000 | 60-65 (old) | male | CellRanger | Intercostal muscle |
| 502B/42 |  | Kedlian et al. 2024 | single nuclei | Illumina HiSeq 4000/NovaSeq 6000 | 60-65 (old) | female | CellRanger | Intercostal muscle |
| 500C/43 |  | Kedlian et al. 2024 | single nuclei | Illumina HiSeq 4000/NovaSeq 6000 | 60-65 (old) | male | CellRanger | Intercostal muscle |
| 343B/44 |  | Kedlian et al. 2024 | single cell,single nuclei | Illumina HiSeq 4000/NovaSeq 6000 | 70-75 (old) | female | CellRanger | Intercostal muscle |
| 367C/45 |  | Kedlian et al. 2024 | single cell,single nuclei | Illumina HiSeq 4000/NovaSeq 6000 | 65-70 (old) | male | CellRanger | Intercostal muscle |
| 339C/46 |  | Kedlian et al. 2024 | single cell,single nuclei | Illumina HiSeq 4000/NovaSeq 6000 | 70-75 (old) | female | CellRanger | Intercostal muscle |
| 640C/47 |  | Kedlian et al. 2024 | single cell | Illumina HiSeq 4000/NovaSeq 6000 | 70-75 (old) | female | CellRanger | Intercostal muscle |
| YM1/48 |  | Lai et al. 2024 | scRNA-seq/snRNA-seq | DIPSEQ T1 | 15 | Male | CellRanger | Semitendinosus |
| P26/49 |  | Lai et al. 2024 | snRNA-seq/snATAC-seq | DIPSEQ T1 | 18 | Female | CellRanger | Semitendinosus |
| YM4/50 |  | Lai et al. 2024 | scRNA-seq/snRNA-seq/snATAC-seq | DIPSEQ T1 | 18 | Male | CellRanger | Semitendinosus |
| P32/51 |  | Lai et al. 2024 | - | DIPSEQ T1 | 25 | Female | CellRanger | Semitendinosus |
| YM2/52 |  | Lai et al. 2024 | snRNA-seq/scATAC-seq/snATAC-seq | DIPSEQ T1 | 34 | Female | CellRanger | Semitendinosus |
| YM3/53 |  | Lai et al. 2024 | scRNA-seq/snRNA-seq/snATAC-seq | DIPSEQ T1 | 34 | Male | CellRanger | Gracilis |
| P13/54 |  | Lai et al. 2024 | snRNA-seq | DIPSEQ T1 | 38 | Male | CellRanger | Gluteus medius |
| P1/55 |  | Lai et al. 2024 | - | DIPSEQ T1 | 39 | Male | CellRanger | Vastus lateralis |
| P11/56 |  | Lai et al. 2024 | - | DIPSEQ T1 | 43 | Female | CellRanger | Pectoralis major |
| P5/57 |  | Lai et al. 2024 | snRNA-seq/snATAC-seq | DIPSEQ T1 | 45 | Male | CellRanger | Tibialis anterior |
| P25/58 |  | Lai et al. 2024 | - | DIPSEQ T1 | 45 | Male | CellRanger | Biceps brachi |
| P9/59 |  | Lai et al. 2024 | - | DIPSEQ T1 | 46 | Male | CellRanger | Vastus lateralis |
| P18/60 |  | Lai et al. 2024 | - | DIPSEQ T1 | 74 | Male | CellRanger | Gluteus medius |
| P29/61 |  | Lai et al. 2024 | snRNA-seq/snATAC-seq | DIPSEQ T1 | 77 | Male | CellRanger | Gluteus medius |
| OM2/62 |  | Lai et al. 2024 | scRNA-seq/snRNA-seq/scATAC-seq/snATAC-seq | DIPSEQ T1 | 79 | Female | CellRanger | Gluteus medius |
| OM4/63 |  | Lai et al. 2024 | scRNA-seq/snRNA-seq/ | DIPSEQ T1 | 79 | Male | CellRanger | Gluteus medius |
| OM9/64 |  | Lai et al. 2024 | snRNA-seq/snATAC-seq | DIPSEQ T1 | 80 | Female | CellRanger | Gluteus medius |
| OM6/65 |  | Lai et al. 2024 | snRNA-seq/snATAC-seq | DIPSEQ T1 | 80 | Female | CellRanger | Gluteus medius |
| OM7/66 |  | Lai et al. 2024 | snRNA-seq/snATAC-seq | DIPSEQ T1 | 82 | Male | CellRanger | Gluteus medius |
| OM5/67 |  | Lai et al. 2024 | scRNA-seq/snRNA-seq/snATAC-seq | DIPSEQ T1 | 82 | Male | CellRanger | Gluteus medius |
| OM1/68 |  | Lai et al. 2024 | scRNA-seq/snRNA-seq/scATAC-seq/snATAC-seq | DIPSEQ T1 | 82 | Female | CellRanger | Gluteus medius |
| OM8/69 |  | Lai et al. 2024 | snRNA-seq | DIPSEQ T1 | 84 | Female | CellRanger | Gluteus medius |
| OM3/70 |  | Lai et al. 2024 | scRNA-seq/snRNA-seq/scATAC-seq/snATAC-seq | DIPSEQ T1 | 84 | Male | CellRanger | Gluteus medius |
| P4/71 |  | Lai et al. 2024 | - | DIPSEQ T1 | 84 | Female | CellRanger | Gluteus medius |
| P23/72 |  | Lai et al. 2024 | snRNA-seq/snATAC-seq | DIPSEQ T1 | 85 | Male | CellRanger | Gluteus medius |
| P17/73 |  | Lai et al. 2024 | snRNA-seq/snATAC-seq | DIPSEQ T1 | 88 | Male | CellRanger | Vastus medialis |
| P28/74 |  | Lai et al. 2024 | - | DIPSEQ T1 | 88 | Male | CellRanger | Gluteus medius |
| P21/75 |  | Lai et al. 2024 | snRNA-seq/snATAC-seq | DIPSEQ T1 | 89 | Female | CellRanger | Gluteus medius |
| P10/76 |  | Lai et al. 2024 | - | DIPSEQ T1 | 90 | Female | CellRanger | Gluteus medius |
| P3/77 |  | Lai et al. 2024 | snRNA-seq | DIPSEQ T1 | 92 | Female | CellRanger | Gluteus medius |
| P27/78 |  | Lai et al. 2024 | snRNA-seq | DIPSEQ T1 | 99 | Female | CellRanger | Gluteus medius |
| GSM4272893/ 79 | SRR10897760 | Micheli et al. 2020 | single cell | Illumina NextSeq 500 | 71 | Male | CellRanger | Right serratus |
| GSM4272894/80 | SRR10897761 | Micheli et al. 2020 | single cell | Illumina NextSeq 500 | 74 | Male | CellRanger | Flexor hallucis longus |
| GSM4272895/81 | SRR10897762 | Micheli et al. 2020 | single cell | Illumina NextSeq 500 | 54 | Male | CellRanger | Orbicularis oculi |
| GSM4272896/82 | SRR10897763 | Micheli et al. 2020 | single cell | Illumina NextSeq 500 | 74 | Male | CellRanger | Eye lid |
| GSM4272897/83 | SRR10897764 | Micheli et al. 2020 | single cell | Illumina NextSeq 500 | 77 | Female | CellRanger | Trapezius |
| GSM4272898/84 | SRR10897765 | Micheli et al. 2020 | single cell | Illumina NextSeq 500 | 54 | Male | CellRanger | Right external oblique |
| GSM4272899/85 | SRR10897766 | Micheli et al. 2020 | single cell | Illumina NextSeq 500 | 70 | Male | CellRanger | Flexor hallucis longus |
| GSM4272900/86 | SRR10897767 | Micheli et al. 2020 | single cell | Illumina NextSeq 500 | 43 | Male | CellRanger | Left vastus lateralis |
| GSM4272901/87 | SRR10897768 | Micheli et al. 2020 | single cell | Illumina NextSeq 500 | 62 | Female | CellRanger | Left external oblique |
| GSM4272902/88 | SRR10897769 | Micheli et al. 2020 | single cell | Illumina NextSeq 500 | 81 | Female | CellRanger | Left recuts abdominus |

**Supplemental Table 1:** **Overview of all individual files/samples included in the study.** The table indicates the ID as used in our atlas as well as the online identifier used to retrieve the file if available. The study of origin as well as the dataset modality, the sequencing modality, age and biological sex are indicated. The sex is indicated as reported in the original study. The preprocessing pipeline is indicated as well as the muscle of origin. ***Abbreviations:*** *sn, single nuclei.*

**Supplemental Figure 2**

**Supplemental Figure 2A:** Benchmarking of integration algorithms. We compared four integration methods – Harmony, reciprocal principal component analysis (RPCA), canonical correlation analysis (CCA), and single-cell variational inference (scVI) – to evaluate their performance in constructing an atlas from multi-study single-cell and single-nucleus RNA-seq data. Integration quality was assessed by each method’s ability to remove batch effects from the originating study (indicated as “Origin” above) or from the studied system (cells or nuclei, indicated as “batch” above), as well as by their capacity to maintain clear separation between cell populations defined by a set of canonical marker genes: MYH1 (Type II myofibers), PAX7 (muscle stem cells, MuSCs), PTPRC/CD45 (immune cells), and COL1A2 (fibro-adipogenic progenitors, FAPs). We evaluated each algorithm on batch removal as well as the preservation of distinct cell populations. All algorithms were applied to the same pre-processed dataset. For RPCA, we compared different integration strength with k.anchor set to 5, 10 and 20. Above, k.anchor = 10 is shown. Lower or higher values did not provide improved results. Overall, RPCA yielded robust integration across most populations but failed to effectively merge cells and nuclei within the mononuclear compartments. CCA tended to overcorrect, as previously reported 1,2, merging biologically distinct cell types. Harmony achieved the best balance, enabling batch removal while preserving distinct cellular identities (see Fig. 1). Integration with scVI was prevented by the lack of raw count data in some studies, leading to spurious and unreliable clustering results.

**Supplemental Figure 2B:** As UMAP parameters may affect the visual presentation, we have added an overview of embeddings generated using different parameter settings. The overall cellular organization remained stable across these settings, supporting the robustness of the embedding structure. ***Abbreviations:*** *CCA*; *Canonical Correlation Analysis. RPCA, Reciprocal PCA; UMAP, Uniform manifold approximation and projection*

**Supplemental Table 2**

| **Method** | **Batch ASW** | **Graph connectivity** | **CiLISI** | **Cell-type ASW** |
| --- | --- | --- | --- | --- |
| Harmony | 0.85 | 0.95 | 0.89 | 0.92 |
| CCA | 0.77 | 0.94 | 0.82 | 0.91 |
| RPCA | 0.79 | 0.93 | 0.81 | 0.87 |

**Supplemental Table 2: Comparison of integration statistics between methods.** To provide an unbiased assessment, we additionally ranked each integration algorithm using the scIB framework. Based on the metrics reported below, Harmony achieved the highest overall score, further supporting its selection for downstream analyses. The following values are indicated: Batch ASW, batch-adjusted silhouette width; higher values indicate better mixing of cells across batches. Graph connectivity quantifies whether cells of the same biological population remain connected after integration; higher values indicate better preservation of within-cell-type structure. CiLISI, cell-type-aware Local Inverse Simpson’s Index; higher values indicate improved batch mixing while accounting for biological identity. Cell-type ASW, cell-type silhouette width; higher values indicate stronger separation of biologically distinct cell populations. Overall, all three methods performed in a highly similar range, indicating no relevant differences in integration quality. Harmony showed a small overall advantage, consistent with our manual inspection based on canonical marker genes.

**Supplemental Figure 3**

**Supplemental Figure 3:** **Cell type prediction using available annotation tools.** **(A+B)** CHETAH and SingleR were used to predict the label for each cell type for each cell/nuclei in the dataset. The Human Primary Cell Atlas (HPCA) was set as a reference. The bar plots show the predicted cell-type levels as percentages of each cell-type cluster in our atlas. ***Abbreviations:*** *BM, bone marrow; CMP, common myeloid progenitor; DC, dendritic cell; FAP, fibro-adipogenic progenitor; GMP, granulocyte-monocyte progenitor; HSC; hematopoietic stem cell; MF, myofiber; MSC, mesenchymal stem cell; MuSC, muscle stem cell; UMAP, Uniform manifold approximation and projection; sc, single cell; sn, single nuclei.*

**Supplemental Figure 4**

**Supplemental Figure 4:** **An annotation tool for skeletal muscle.** **(A)** Validation of the marker-based annotation tool using ranked marker gene lists from an independent snRNA-seq dataset from patients with inflammatory muscle diseases. Each external marker list was scored against the atlas-derived reference cell types. The heatmap shows the top predicted identity for each external cell type. **(B)** Overlap between the external marker gene lists and the reference marker sets. The overlap indicates the percentage of marker genes from the external marker gene list detected in our reference markers per cell type. **(C+D)** Comparison with either CHETAH or SingleR for either cell type. ***Abbreviations:*** *FAP, fibro-adipogenic progenitor; MF, myofiber; MuSC, muscle stem cell; naFb, nerve-associated fibroblasts; NMJ, neuromuscular junction.*

**Supplemental Figure 5**

**Supplemental Figure 5: Top FAP marker genes. (A)** Dot plot showing the expression of the top 10 FAP marker genes across cell types. Dot size represents the percentage of cells expressing each gene, and colour indicates scaled average expression. **(B)** Receiver operating characteristic (ROC) analysis of the same top 10 FAP marker genes for distinguishing FAPs from all other cell types. Area under the curve (AUC) values are shown for each marker gene. ***Abbreviations:*** *AUC, area under the curve; FAP, fibro-adipogenic progenitor; ROC, receiver operating characteristic.*

**Supplemental Figure 6**

**Supplementary Fig. 6: NOVA1 expression in skeletal muscle reference datasets.**
(A) UMAP visualization of the human skeletal muscle reference dataset, with nuclei annotated according to the provided cell-type labels of the original study.
(B) Feature plot showing *NOVA1* expression across nuclei. Each dot is one nucleus, coloured by *NOVA1* expression. (C) UMAP visualization of the mouse skeletal muscle reference dataset, with nuclei annotated according to labels of the original study.
(D) Feature plot showing *Nova1* expression across nuclei. Each dot is one nucleus, coloured by *Nova1* expression. ***Abbreviations****: UMAP, uniform manifold approximation and projection.*

**Supplemental Figure 7**

**Supplemental Figure 7:** **Co-expression network of NOVA1.** **(A)** Heatmap demonstrating co-expressed genes for NOVA1. NOVA1 expression was partitioned into 50 bins. For each gene we computed the mean log-expression within each NOVA1 bin. Gene inclusion required detection in ≥ 0.5% of FAPs and non-zero variance across bins. Gene association with NOVA1 was quantified by Pearson correlation between the gene’s mean profile and the NOVA1 means. **(B)** Gene set enrichment analysis (GSEA) for the GO Biological Process (BP) dataset. Pre-ranked enrichment was performed with fgseaMultilevel. ***Abbreviations:*** *FDR, false-discovery rate.*

**Supplemental Figure 8**

**Supplemental Figure 8:** **Expression of myogenic marker genes.** **(A-D)** Expression of each marker gene per nuclei overlaid as a feature plot onto the UMAP. The expression is normalized and colour-coded. Nuclei that do not express the respective gene are grey. ***Abbreviations:*** *UMAP, Uniform manifold approximation.*

**Supplemental Figure 9**

**Supplemental Figure 9:** **Protocadherin expression in myogenic cells.** Feature plots indicating the expression of *MYH1*, *MYH2*, *MYH7*, as well as exemplary protocadherin genes *PCDHGA3*, *PCDHGA6* and *PCDHGA9*. MYH7 marks nuclei belonging to type I myofibers and MYH1/MYH2 mark nuclei belonging to type II myofibers. Each dot is one nucleus, coloured by the normalized expression of the indicated gene. For all plots, genes with a normalized expression below 2 are not shown. ***Abbreviations:*** *MYH, myosin heavy chain; PCDH, protocadherin.*

**Supplemental Figure 10**

**Supplemental Figure 10:** **Cross-validation of protocadherin expression.** Analysis of an external RNA-seq dataset. Raw data were downloaded from the European Genome-phenome Archive (EGA) and processed. We generated pseudobulk expressions for each MF type within the original 1000-fiber dataset. The original data was acquired from 14 individual participants. Data was aggregated and analysed per participant. We searched all genes for genes starting with PCDH and calculated their normalized expression and protein abundance levels. **(A)** Normalized gene expression for the indicated genes. **(B)** Normalized protein abundance for the indicated protein. Groups were compared by the two-sided Student`s T test. Each dot represents one participant from the original study. n = 13 for type I MF and n = 15 for type II MF. 15 Error bars indicate the standard deviation. * p ≤ 0.05. ***Abbreviations:*** *MF, myofiber; PCDH, protocadherin.*
