# Supplementary material for: An integrated reference atlas of human skeletal muscle
Source: eBioMedicine. 2026 Aug 31;131:106465. doi: 10.1016/j.ebiom.2026.106465 (PMC13562069; doi:10.1016/j.ebiom.2026.106465)
Supplement: Supplementary file 2 [file mmc5.docx]

**An Integrated Reference Atlas of Human Skeletal Muscle**

**Supplementary Files Captions**

The location and caption are listed below.

**Supplementary Figures Page 2**

**Supplemental Figure 1:** Mitochondrial reads across studies and cell types. Violin plots indicating the percentage of mitochondrial reads for each study (A) and each cell type (B). The box plot indicates the median and error bars indicate the minimum and maximum values.

**Supplementary Figures Page 3 to 7**

**Supplemental Table 1:** Overview of all individual files/samples included in the study. The table indicates the ID as used in our atlas as well as the online identifier used to retrieve the file if available. The study of origin as well as the dataset modality, the sequencing modality, age and biological sex are indicated. The sex is indicated as reported in the original study. The preprocessing pipeline is indicated as well as the muscle of origin. *Abbreviations: sn, single nuclei.*

**Supplementary Figures Page 8 to 9**

**Supplemental Figure 2A:** Benchmarking of integration algorithms. We compared four integration methods – Harmony, reciprocal principal component analysis (RPCA), canonical correlation analysis (CCA), and single-cell variational inference (scVI) – to evaluate their performance in constructing an atlas from multi-study single-cell and single-nucleus RNA-seq data. Integration quality was assessed by each method’s ability to remove batch effects from the originating study (indicated as “Origin” above) or from the studied system (cells or nuclei, indicated as “batch” above), as well as by their capacity to maintain clear separation between cell populations defined by a set of canonical marker genes: MYH1 (Type II myofibers), PAX7 (muscle stem cells, MuSCs), PTPRC/CD45 (immune cells), and COL1A2 (fibro-adipogenic progenitors, FAPs). We evaluated each algorithm on batch removal as well as the preservation of distinct cell populations. All algorithms were applied to the same pre-processed dataset. For RPCA, we compared different integration strength with k.anchor set to 5, 10 and 20. Above, k.anchor = 10 is shown. Lower or higher values did not provide improved results. Overall, RPCA yielded robust integration across most populations but failed to effectively merge cells and nuclei within the mononuclear compartments. CCA tended to overcorrect, as previously reported 1,2, merging biologically distinct cell types. Harmony achieved the best balance, enabling batch removal while preserving distinct cellular identities (see Fig. 1). Integration with scVI was prevented by the lack of raw count data in some studies, leading to spurious and unreliable clustering results. **Supplemental Figure 2B:** As UMAP parameters may affect the visual presentation, we have added an overview of embeddings generated using different parameter settings. The overall cellular organization remained stable across these settings, supporting the robustness of the embedding structure. ***Abbreviations:*** *CCA*; *Canonical Correlation Analysis. RPCA, Reciprocal PCA; UMAP, Uniform manifold approximation and projection*

**Supplementary Figures Page 10**

**Supplemental Table 2:** Comparison of integration statistics between methods. To provide an unbiased assessment, we additionally ranked each integration algorithm using the scIB framework. Based on the metrics reported below, Harmony achieved the highest overall score, further supporting its selection for downstream analyses. The following values are indicated: Batch ASW, batch-adjusted silhouette width; higher values indicate better mixing of cells across batches. Graph connectivity quantifies whether cells of the same biological population remain connected after integration; higher values indicate better preservation of within-cell-type structure. CiLISI, cell-type-aware Local Inverse Simpson’s Index; higher values indicate improved batch mixing while accounting for biological identity. Cell-type ASW, cell-type silhouette width; higher values indicate stronger separation of biologically distinct cell populations. Overall, all three methods performed in a highly similar range, indicating no relevant differences in integration quality. Harmony showed a small overall advantage, consistent with our manual inspection based on canonical marker genes.

**Supplementary Figures Page 11**

**Supplemental Figure 3:** **Cell type prediction using available annotation tools.** **(A+B)** CHETAH and SingleR were used to predict the label for each cell type for each cell/nuclei in the dataset. The Human Primary Cell Atlas (HPCA) was set as a reference. The bar plots show the predicted cell-type levels as percentages of each cell-type cluster in our atlas. Abbreviations: BM, bone marrow; CMP, common myeloid progenitor; DC, dendritic cell; FAP, fibro-adipogenic progenitor; GMP, granulocyte-monocyte progenitor; HSC; hematopoietic stem cell; MF, myofiber; MSC, mesenchymal stem cell; MuSC, muscle stem cell; UMAP, Uniform manifold approximation and projection; sc, single cell; sn, single nuclei.

**Supplemental File 1 (Excel File “Suppl_File_1.xlsx):**

**Supplemental File 1:** Full list of all differentially expressed marker genes as referenced in Figure 2. The table indicates the P-value (p_val), the average log 2 fold change (avg_log2FC), the percentage of cells expressing a given gene (pct.1 and pct.2) the adjusted P-value (p_val_adj) and the cluster and gene names.

**Supplementary Figures Page 12**

**Supplemental Figure 4:** An annotation tool for skeletal muscle. (A) Validation of the marker-based annotation tool using ranked marker gene lists from an independent snRNA-seq dataset from patients with inflammatory muscle diseases. Each external marker list was scored against the atlas-derived reference cell types. The heatmap shows the top predicted identity for each external cell type. (B) Overlap between the external marker gene lists and the reference marker sets. The overlap indicates the percentage of marker genes from the external marker gene list detected in our reference markers per cell type. (C+D) Comparison with either CHETAH or SingleR for either cell type. Abbreviations: FAP, fibro-adipogenic progenitor; MF, myofiber; MuSC, muscle stem cell; naFb, nerve-associated fibroblasts; NMJ, neuromuscular junction.

**Supplementary Figures Page 13**

**Supplemental Figure 5:** Top FAP marker genes. (A) Dot plot showing the expression of the top 10 FAP marker genes across cell types. Dot size represents the percentage of cells expressing each gene, and colour indicates scaled average expression. (B) Receiver operating characteristic (ROC) analysis of the same top 10 FAP marker genes for distinguishing FAPs from all other cell types. Area under the curve (AUC) values are shown for each marker gene. Abbreviations: AUC, area under the curve; FAP, fibro-adipogenic progenitor; ROC, receiver operating characteristic.

**Supplementary Figures Page 14**

**Supplementary Fig. 6:** NOVA1 expression in skeletal muscle reference datasets.
(A) UMAP visualization of the human skeletal muscle reference dataset, with nuclei annotated according to the provided cell-type labels of the original study.
(B) Feature plot showing NOVA1 expression across nuclei. Each dot is one nucleus, coloured by NOVA1 expression. (C) UMAP visualization of the mouse skeletal muscle reference dataset, with nuclei annotated according to labels of the original study.
(D) Feature plot showing Nova1 expression across nuclei. Each dot is one nucleus, coloured by Nova1 expression. Abbreviations: UMAP, uniform manifold approximation and projection.

**Supplementary Figures Page 15**

**Supplemental Figure 7:** Co-expression network of NOVA1. (A) Heatmap demonstrating co-expressed genes for NOVA1. NOVA1 expression was partitioned into 50 bins. For each gene we computed the mean log-expression within each NOVA1 bin. Gene inclusion required detection in ≥ 0.5% of FAPs and non-zero variance across bins. Gene association with NOVA1 was quantified by Pearson correlation between the gene’s mean profile and the NOVA1 means. (B) Gene set enrichment analysis (GSEA) for the GO Biological Process (BP) dataset. Pre-ranked enrichment was performed with fgseaMultilevel. Abbreviations: FDR, false-discovery rate.

**Supplemental File 2 (Excel File “Suppl_File_2.xlsx)**

**Supplemental File 2:** Statistics for the regression analysis and binning for *NOVA1* expression in the FAP cell type. **A: NOVA1_Bins**. Table indicating the individual bins used for regression analysis, their mean expression for *NOVA1* and the normalized (z-scaled) *NOVA1* expression per bin. **B: Gene_Stats.** Table indicating each gene in the regression analysis with its r- and t-statistics, the corresponding P-value and the adjusted P-value. **C: Heatmap_Z.** Table indicating the normalized (z-scaled) score per bin for each gene in the regression analysis. D: GO_BP_fgsea. Results of the GO-BP gene set enrichment analysis. Genes were ranked by their regression coefficients and analysed with the GO-BP database. The table indicates the pathway with the GO identifier, the P-value and adjusted P-value, the enrichment score (ES) and normalized enrichment score (NES), the gene set size (size), the contributing genes (leadingEdge), and the term name (term).

**Supplementary Figures Page 16**

**Supplemental Figure 8:** Expression of myogenic marker genes. (A-D) Expression of each marker gene per nuclei overlaid as a feature plot onto the UMAP. The expression is normalized and colour-coded. Nuclei that do not express the respective gene are grey. Abbreviations: UMAP, Uniform manifold approximation.

**Supplemental File 3 (Excel File “Suppl_File_3.xlsx”)**

**Supplemental File 3:** Output of Genes2Genes analysis showing the alignment for individual genes as shown in Figure 5B, C and D. The table indicates the alignment similarity percentage for each analysed gene, the optimal alignment costs and the log2 fold change between type 1 and type 2 myofibers.

**Supplementary Figures Page 17**

**Supplemental Figure 9:** Protocadherin expression in myogenic cells. Feature plots indicating the expression of MYH1, MYH2, MYH7, as well as exemplary protocadherin genes PCDHGA3, PCDHGA6 and PCDHGA9. MYH7 marks nuclei belonging to type I myofibers and MYH1/MYH2 mark nuclei belonging to type II myofibers. Each dot is one nucleus, coloured by the normalized expression of the indicated gene. For all plots, genes with a normalized expression below 2 are not shown. Abbreviations: MYH, myosin heavy chain; PCDH, protocadherin.

**Supplementary Figures Page 17**

**Supplemental Figure 10:** Cross-validation of protocadherin expression. Analysis of an external RNA-seq dataset. Raw data were downloaded from the European Genome-phenome Archive (EGA) and processed. We generated pseudobulk expressions for each MF type within the original 1000-fiber dataset. The original data was acquired from 14 individual participants. Data was aggregated and analysed per participant. We searched all genes for genes starting with PCDH and calculated their normalized expression and protein abundance levels. (A) Normalized gene expression for the indicated genes. (B) Normalized protein abundance for the indicated protein. Groups were compared by the two-sided Student`s T test. Each dot represents one participant from the original study. n = 13 for type I MF and n = 15 for type II MF. 15 Error bars indicate the standard deviation. * p ≤ 0.05. Abbreviations: MF, myofiber; PCDH, protocadherin.
